# Uniaxial versus biaxial character of nematic equilibria in three dimensions

**DOI:** 10.1007/s00526-017-1142-8

**Published:** 2017-04-04

**Authors:** Duvan Henao, Apala Majumdar, Adriano Pisante

**Affiliations:** 1 Department of Mathematical Sciences, University of Bath, Bath, BA2 7AY United Kingdom; 2 Dipartimento di Matematica “G. Castelnuovo”, Sapienza Università di Roma, 00185 Rome, Italy; 3 Facultad de Matemáticas, Pontificia Universidad Católica de Chile, Casilla 306, Correo 22, Santiago, Chile

## Abstract

We study global minimizers of the Landau–de Gennes (LdG) energy functional for nematic liquid crystals, on arbitrary three-dimensional simply connected geometries with topologically non-trivial and physically relevant Dirichlet boundary conditions. Our results are specific to an asymptotic limit coined in terms of a dimensionless temperature and material-dependent parameter, *t* and some constraints on the material parameters, and we work in the $t\rightarrow \infty$ limit that captures features of the widely used Lyuksyutov constraint (Kralj and Virga in J Phys A 34:829–838, 2001). We prove (i) that (re-scaled) global LdG minimizers converge uniformly to a (minimizing) limiting harmonic map, away from the singular set of the limiting map; (ii) we have points of maximal biaxiality and uniaxiality near each singular point of the limiting map; (iii) estimates for the size of “strongly biaxial” regions in terms of the parameter *t*. We further show that global LdG minimizers in the restricted class of uniaxial $\mathbf{Q}$-tensors cannot be stable critical points of the LdG energy in this limit.

## Introduction

Nematic liquid crystals (LCs) are anisotropic liquids with long-range orientational order i.e. the constituent rod-like molecules have full translational freedom but align along certain locally preferred directions [11, 38]. The existence of distinguished directions renders nematics sensitive to light and external fields leading to unique electromagnetic, optical and rheological properties [11, 16, 30]. The analysis of nematic spatio–temporal patterns is a fascinating source of problems for mathematicians, physicists and engineers alike, especially in the context of defects or material singularities [13, 16].

In recent years, mathematicians have turned to the analysis of the celebrated Landau–de Gennes (LdG) theory for nematic liquid crystals, particularly in various asymptotic limits: see for example [8, 10, 15, 24] which is not an exhaustive list but are directly relevant to our paper. The LdG theory is a variational theory with an associated energy functional, defined in terms of a macroscopic order parameter, known as the $\mathbf{Q}$-tensor order parameter [11, 21, 38]. Mathematically speaking, the LdG $\mathbf{Q}$-tensor is a symmetric traceless $3 \times 3$ matrix. The LdG energy typically comprises an elastic energy, convex in ${{\nabla }}\mathbf{Q}$ with several elastic constants, and a non-convex bulk potential, $f_B$ defined in terms of the temperature and the eigenvalues of $\mathbf{Q}$-tensor [27]. With the one-constant approximation for the elastic energy density, the LdG energy functional has a similar structure to the Ginzburg–Landau (GL) functional for superconductivity [5, 9, 28] and we can borrow several ideas from GL theory to make qualitative predictions about global energy minimizers, at least away from singularities. However, there is an important distinction between the GL and LdG theories. In the GL-framework, researchers study maps, $\mathbf {u}: {\mathbb {R}}^d \rightarrow {\mathbb {R}}^m$, $d,m=2,3$ (see e.g. [5, 25, 31]), whereas the LdG variable is a five-dimensional map, $\mathbf{Q}: {\mathbb {R}}^3 \rightarrow {\mathbb {R}}^5$. A uniaxial $\mathbf{Q}$-tensor has two degenerate non-zero eigenvalues and hence, three degrees of freedom and in this case, there is broader scope for transferable methodologies (see for example, [15]). A biaxial $\mathbf{Q}$-tensor has three distinct eigenvalues with five degrees of freedom and maximal biaxiality corresponds to a vanishing eigenvalue. There are a plethora of open questions about how the two extra degrees of freedom manifest in the mathematics and physics of biaxial systems.

We re-visit questions related to the uniaxial versus biaxial structure of global LdG minimizers. We work with the simplest form of the LdG energy:

1 $$\begin{aligned} I\left[ \mathbf{Q}\right] : = \int _{\Omega } \frac{L}{2} \left| {{\nabla }}\mathbf{Q}\right| ^2 + f_B\left( \mathbf{Q}\right) ~dV \end{aligned}$$

with the one-constant Dirichlet elastic energy density and a quartic form of the bulk potential

2 $$\begin{aligned} f_B\left( \mathbf{Q}\right) : =\frac{A}{2}{{\text {tr}}}\mathbf{Q}^2 - \frac{B}{3}{\text {tr}}\mathbf{Q}^3 + \frac{C}{4}\left( \text {tr}\mathbf{Q}^2 \right) ^2 \end{aligned}$$

where *A* is the re-scaled temperature, *B* and *C* are material-dependent constants [27]. The bulk potential, $f_B$, is minimized by uniaxial tensors of a constant norm for low temperatures i.e. for $A<0$, $f_B$ is minimized on the set of uniaxial tensors $\mathbf{Q}_{\min }$, with $|\mathbf{Q}_{\min }|^2 = \frac{2}{3} s_+^2$, and $s_+$ can be explicitly worked out in terms of *A*, *B*, *C* [4, 22, 27]. We work with large domains, $\Omega \subset {\mathbb {R}}^3$, whose characteristic length *D* is much larger than the temperature-dependent biaxial correlation length, $\xi _b = \sqrt{\frac{L}{B s_+}}$ [17, 18] i.e. we assume that $\frac{D}{\xi _b} \gg 1$. We first present some heuristics (known in the literature) to motivate our rigorous results. Let $I_{eq}$ denote the LdG energy of a bulk equilibrium i.e. a spatially homogeneous state that minimizes $f_B$ at a given $A<0$. Scaling the $\mathbf{Q}$-tensors according to $\mathbf{Q}= \sqrt{\frac{2}{3}}s_+ \bar{\mathbf{Q}}$, we obtain

$$\begin{aligned} I\left[ \mathbf{Q}\right] - I_{eq}: = s_+^2 \int _{\Omega } \frac{L}{2}\left| {{\nabla }}\bar{\mathbf{Q}} \right| ^2 + \frac{\left| A\right| }{6}\left( 1 - \left| \bar{\mathbf{Q}} \right| ^2 \right) ^2 + \frac{B s_+}{54}\left( 1 + 3 \left| \bar{\mathbf{Q}}\right| ^4 - 4\sqrt{6} {\text {tr}}\bar{\mathbf{Q}}^3 \right) ~dV. \end{aligned}$$

In the language of length scales commonly adopted in the literature [17, 18], the uniaxial correlation length, $\xi _u \sim \sqrt{\frac{L}{|A|}}$, and if we work with $\xi _u \ll \xi _b \ll D$, then the expression for $I[\mathbf{Q}] - I_{eq}$ above suggests that it is energetically preferable to have $|\bar{\mathbf{Q}}|^2 =1$ almost everywhere and to overcome topological frustration by allowing for biaxiality within small regions of size proportional to $\xi _b$. In other words, we would expect LdG minimizers in this regime to have constant norm and defect cores to have biaxial structures with size dictated by $\xi _b$.

We work with a dimensionless temperature and material-dependent parameter

3 $$\begin{aligned} t = \frac{27 \left| A\right| C}{B^2}. \end{aligned}$$

Let $h_+ = \frac{3 + \sqrt{9 + 8t}}{4}$ and a direct calculation shows that $s_+ = \frac{B}{3C} h_+$. Then the condition $\xi _u \ll \xi _b$ is equivalent to $t\gg h_+$ or $t\gg 1$, which in turn is equivalent to $\left| T - T_{IN} \right| \gg \left| T_{IN} - T_* \right|$ where $T_{IN}$ is the isotropic-nematic transition temperature and $T_*$ is another critical temperature [17, 18]. In the literature, this is referred to as being “deep in the nematic phase” and this separation of scales can be achieved if *B* is an order of magnitude smaller than |*A*| and *C* (also see [14]). This assumption can be true for some commonly used liquid crystal materials [18]. Indeed, this assumption forms the basis of the popular *Lyuksyutov* constraint in the literature where authors work with $\mathbf{Q}$-tensors of a fixed norm [17, 18] i.e.

$$\begin{aligned} \left| \mathbf{Q}\right| ^2 = \frac{\left| A\right| }{C} \end{aligned}$$

for sufficiently negative $A<0$.

In what follows, we assume that $\xi _u \ll \xi _b \ll D$ and perform an asymptotic analysis of global LdG minimizers in the $t \rightarrow \infty$ limit, for a fixed value of the ratio, $\frac{D}{\xi _0}$, where $\xi _0$ is a characteristic material-dependent length scale often referred to as the bare biaxial correlation length [17]. We note that *t* cannot be unbounded but as with any asymptotic analysis, it is reasonable to expect that an analysis in the $t \rightarrow \infty$ limit provides at least qualitative information for physically relevant large values of *t*, as have often been quoted in the literature. We can interpret this asymptotic limit in at least two different ways. One interpretation is to keep *B*, *C*, *L* fixed and vary the temperature, in terms of *A*. In the “deep nematic phase”, one could argue that the re-scaled temperature, |*A*|, is larger than *B* and *C* and therefore, *t* is large (say $t \sim 10$; see [14]) and we might hope that our asymptotic analysis sheds qualitative insight into equilibrium properties for large but bounded values of *t*. For example, the celebrated biaxial torus solution on a three-dimensional droplet has been numerically reported in [14, 17, 18] for large values of *t* and our rigorous results reproduce some qualitative features of the biaxial torus in this asymptotic limit. In [14], the authors plot the eigenvalues of the biaxial torus solution away from the droplet centre and they find that the solution is uniaxial with positive order parameter at the droplet centre, followed by a torus of maximal biaxiality which contains an uniaxial ring of negative scalar order parameter; the biaxial torus then relaxes to match the imposed uniaxial Dirichlet radial boundary conditions. The uniaxial ring of negative order parameter contained within the biaxial torus is often referred to as a defect. Indeed private communication [16, 33] suggests that experimentalists have now observed the biaxial torus solution. We rigorously prove the co-existence of both maximal biaxiality and uniaxiality near each singular point (to be interpreted appropriately) of a global LdG minimizer on an arbitrary three-dimensional domain with uniaxial Dirichlet boundary conditions, and whilst this does not recover the structure of the biaxial torus solution, this is consistent with the picture of a torus of maximal biaxiality that contains a uniaxial ring of negative scalar order parameter.

We note that the eigenvalues of the minimizers of the bulk potential $f_B$ in (2) respect physical bounds if and only if [22, 38]

4 $$\begin{aligned} \frac{1}{3}\left( B - 2C\right) \le A \le \frac{B^2}{24 C}. \end{aligned}$$

If one were to work with $A \ll 0$ i.e. with very low temperatures for fixed *B* and *C*, then the physicality constraints on the eigenvalues are violated. In this case, the bulk potential $f_B$ in (2) could be replaced by the Ball–Majumdar potential introduced in [2], the advantage being that the minimizers of the Ball–Majumdar potential respect the physical constraints on the eigenvalues for all temperatures. We expect the analysis in this paper to aid future work on these lines.

The other interpretation is to work with materials such that $B \propto \sqrt{\frac{L B_0}{D^2}}=o(1)$, where $B_0$ is some reference value for *B*, keeping |*A*| and *C* fixed. This condition is compatible with (4) and large values of *t* if one works with materials for which $B = o\left( C \right)$ and $A<0$, $C>0$ are fixed and consistent with (4). In particular, the physicality constraints on the eigenvalues can be respected with such an interpretation of the $t \rightarrow \infty$ limit, for domains with $\xi _u \ll \xi _b \ll D$.

Next, we briefly summarize the main results of this paper. We work with fixed topologically non-trivial Dirichlet boundary conditions, that are minimizers of the potential, $f_B$, for $A<0$. We first prove that global LdG minimizers converge uniformly to a (minimizing) limiting harmonic map, away from the singular set of the limiting harmonic map, in this asymptotic limit. The singular set of a minimizing harmonic map is a finite set of isolated points [6, 34]. The uniform convergence follows from a Bochner-type inequality for the LdG energy density, first derived in [24] in the so-called vanishing elastic constant limit. We have two new cases to consider compared to [24], whilst deriving the Bochner inequality, see Cases II and III of Lemma 3.3, since we are studying a different asymptotic limit. The uniform convergence gives a fairly good picture away from the singular set of the limiting harmonic map. The next step is to adapt arguments from [10] to prove that the norm of a global LdG minimizer converges uniformly to a constant value, everywhere on $\Omega$ (not merely away from singularities). In Theorem 1 (iii), we provide a rigorous proof of the norm convergence in the $t \rightarrow \infty$ limit, yielding a rigorous justification of the common Lyuksyutov constraint in the literature. In this spirit, one may refer to our limit as the *Lyuksyutov limit*. In [10], the authors prove the global LdG minimizers have at least a point of maximal biaxiality, in the $t \rightarrow \infty$ limit, but do not comment on the number or location of such points. We appeal to a topological result in [8] to deduce the existence of at least a point of maximal biaxiality and a point of uniaxiality near each singular point of the limiting map, in the $t \rightarrow \infty$ limit; maximal biaxiality occurs when $\mathbf{Q}$ has a vanishing eigenvalue (see [26, 38]). We do not attempt to prove rigorous results about the Hausdorff measure or dimension of the regions of maximal biaxiality or uniaxiality i.e. do we have a biaxial torus or an uniaxial ring near each defect, since this is outside the scope of this paper. We make the notion of “strongly biaxial” regions in global minimizers more precise by computing estimates for the size of such regions in terms of *t*. The proof depends on scaling and blow-up arguments and well-established results in GL-theory and the theory of harmonic maps (e.g. [6, 34]). We consider all admissible scenarios and exclude all but one scenario, to find that the size of “strongly biaxial” regions scales as $t^{-1/4}$ as $t \rightarrow \infty$. This implies that we only see strong biaxiality inside regions with radius proportional to the biaxial correlation length, $\xi _b$, as would be expected on physical grounds [17, 18] (see Sect. 2 for precise statement).

Our second theorem generalizes the results in [15] to arbitrary 3D geometries with topologically non-trivial Dirichlet conditions. There exists a global LdG energy minimizer in the restricted class of uniaxial $\mathbf{Q}$-tensors, for all material constants and temperatures. We show that these restricted minimizers cannot be stable critical points of the LdG energy for sufficiently large *t* in (3), for fixed *L* in (1). Appealing to topological arguments, we prove that any uniaxial critical point of the LdG energy, subject to the hypotheses of Proposition 2.1, has an isotropic point (with $\mathbf{Q}=0$) near each singular point of the limiting harmonic map, as $t \rightarrow \infty$. We then proceed with a local version of the global analysis in [15], equipped with certain energy quantization results for harmonic maps [6] and blow-up techniques, to deduce the local radial-hedgehog (RH) profile near each isotropic point and the instability of the RH profile for large *t* suffices to conclude the argument. In [20], the author rules out uniaxial critical points in one and two dimensional domains but the RH solution is an example of a semi-explicit uniaxial critical point (excluding an isolated isotropic point) on a 3D droplet and hence, uniaxial critical points in 3D remain of interest.

The paper is organized as follows. In Sect. 2, we review the theoretical background and state our main results. In Sect. 3, we give the proofs and in Sect. 4, we conclude with future perspectives.

## Statement of results

Let $\Omega \subset {\mathbb {R}}^3$ be an arbitrary simply-connected 3D domain with smooth boundary. Let $\mathbb {S}^2$ be the set of unit vectors in $\mathbb {R}^3$ and let $S_0$ denote the set of symmetric, traceless $3\times 3$ matrices i.e.

5 $$\begin{aligned} S_0 = \left\{ \mathbf{Q}\in M^{3\times 3}; \mathbf{Q}_{ij}=\mathbf{Q}_{ji}; \mathbf{Q}_{ii}=0 \right\} , \end{aligned}$$

where $M^{3\times 3}$ is the set of $3\times 3$ matrices. The corresponding matrix norm is defined to be [24]

6 $$\begin{aligned} \left| \mathbf{Q}\right| ^2 = \mathbf{Q}_{ij} \mathbf{Q}_{ij} \quad i,j=1\ldots 3 \end{aligned}$$

and we use the Einstein summation convention throughout the paper.

We work with the Landau–de Gennes (LdG) theory for nematic liquid crystals [11] whereby a LC state is described by a macroscopic order parameter: the $\mathbf{Q}$-tensor order parameter. The $\mathbf{Q}$-tensor is a macroscopic measure of the LC anisotropy. Mathematically, the LdG $\mathbf{Q}$-tensor order parameter is a symmetric, traceless $3\times 3$ matrix in the space $S_0$ in (5). A LC state is said to be (i) isotropic (disordered with no orientational ordering) when $\mathbf{Q}=0$, (ii) uniaxial when $\mathbf{Q}$ has two degenerate non-zero eigenvalues and (iii) biaxial when $\mathbf{Q}$ has three distinct eigenvalues. A uniaxial $\mathbf{Q}$-tensor can be written as

7 $$\begin{aligned} \mathbf {Q}(\mathbf {x}) = s(\mathbf {x}) \left( \mathbf {n}(\mathbf {x}) \otimes \mathbf {n}(\mathbf {x}) - \frac{\mathbf {I}}{3}\right) , \end{aligned}$$

for some $s(\mathbf {x})\in \mathbb {R}$ and some unit-vector $\mathbf {n}(\mathbf {x})\in \mathbb {S}^2$, for a.e. $\mathbf {x} \in \Omega$. We include $s=0$ in our definition although $s=0$ corresponds to the isotropic phase. The unit-vector, $\mathbf {n}$, is the director or equivalently, the single distinguished direction of molecular alignment in the sense that all directions orthogonal to $\mathbf {n}$ are physically equivalent for an uniaxial nematic. We recall the definition of the biaxiality parameter [26, 38],

8 $$\begin{aligned} \beta ^2 = 1 - \frac{6 \left( {{\text {tr}}}\mathbf{Q}^3\right) ^2}{\left| \mathbf{Q}\right| ^6} \in \left[ 0, 1 \right] . \end{aligned}$$

The definition (8) is commonly accepted in the literature and works well to differentiate biaxial phases from uniaxial phases since $\beta ^2=0$ if and only if $\mathbf{Q}$ is uniaxial i.e. if and only if $|\mathbf{Q}|^6 = 6\left( {\text {tr}} \mathbf{Q}^3 \right) ^2$ [4] and we have maximal biaxiality $\beta ^2 =1$ if and only if ${\text {tr}}\mathbf{Q}^3 = 0$ which necessarily requires a vanishing eigenvalue.

The LdG theory is a variational theory and has an associated LdG free energy. The LdG energy density is a nonlinear function of $\mathbf{Q}$ and its spatial derivatives [11, 27]. We work with the simplest form of the LdG energy functional that allows for a first-order nematic-isotropic phase transition and spatial inhomogeneities as shown below [24, 27]:

9 $$\begin{aligned} \mathbf{I_{LG}}\left[ \mathbf{Q}\right] = \int _{\Omega } \frac{L}{2}\left| {{\nabla }}\mathbf{Q}\right| ^2 + f_B\left( \mathbf{Q}\right) ~dV. \end{aligned}$$

Here, $L>0$ is a small material-dependent elastic constant, $|{{\nabla }}\mathbf{Q}|^2 = \mathbf{Q}_{ij,k}\mathbf{Q}_{ij,k}$ (note that $\mathbf{Q}_{ij,k} = \frac{\partial \mathbf{Q}_{ij}}{\partial \mathbf{x}_k}$) with $i,j,k=1,2,3$ is an *elastic energy density* and $f_B:S_0 \rightarrow {\mathbb {R}}$ is the *bulk energy density* that dictates the preferred nematic phase: isotropic/uniaxial/biaxial. For our purposes, we take $f_B$ to be a quartic polynomial in the $\mathbf{Q}$-tensor invariants:

10 $$\begin{aligned} f_B(\mathbf{Q}) = \frac{A(T)}{2}{\text {tr}}\mathbf{Q}^2 - \frac{B}{3}{\text {tr}}\mathbf{Q}^3 + \frac{C}{4}\left( {\text {tr}}\mathbf{Q}^2\right) ^2 \end{aligned}$$

where ${\text {tr}}\mathbf{Q}^3 = \mathbf{Q}_{ij}\mathbf{Q}_{jp}\mathbf{Q}_{pi}$ with $i,j,p=1,2,3$; $A(T) = \alpha (T - T^*)$; $\alpha , B, C>0$ are material-dependent constants, *T* is the absolute temperature and $T^*$ is a characteristic temperature below which the isotropic phase, $\mathbf{Q}=0$, loses its stability [22, 27]. One can readily verify that $f_B$ is bounded from below and attains its minimum on the set of $\mathbf{Q}$-tensors given by Majumdar [22, 23]

11 $$\begin{aligned} \mathbf{Q}_{\min } = \left\{ \mathbf{Q}\in S_0; \mathbf{Q}= s_+ \left( \mathbf{n}\otimes \mathbf{n}- \frac{\mathbf {I}}{3}\right) ,~\mathbf{n}\in \mathbb {S}^2 \right\} , \end{aligned}$$

$\mathbf {I}$ is the $3\times 3$ identity matrix and

12 $$\begin{aligned} s_+ = \frac{B + \sqrt{B^2 + 24 \left| A\right| C}}{4 C}. \end{aligned}$$

The set, (11), is the set of uniaxial $\mathbf {Q}$-tensors with constant order parameter, $s_+$. The physically relevant range is often defined to be $s_+ \in \left( 0, 1 \right)$ [2, 22] and the physicality constraint is clearly violated for $A<0$ and $|A|\gg 1$, for fixed *B* and *C*.

In what follows, we take the Dirichlet boundary condition to be

13 $$\begin{aligned} \mathbf{Q}_{b}(\mathbf{x}) = s_+ \left( \mathbf {n}_b \otimes \mathbf {n}_b - \frac{\mathbf {I}}{3}\right) \end{aligned}$$

for some arbitrary smooth unit-vector field, $\mathbf {n}_b$, with topological degree $d\ne 0$ (see, e.g., [7] and [6] for the definition and the main properties of the topological degree). In particular, this means that $\mathbf{n}_b$ has no continuous $S^2$-valued extension inside $\Omega$. The corresponding admissible space is

14 $$\begin{aligned} {\mathcal {A}}= \left\{ \mathbf{Q}\in W^{1,2}\left( \Omega ;S_0\right) ; \mathbf{Q}= \mathbf{Q}_{b} ~\text {on}~ \partial \Omega \right\} , \end{aligned}$$

where $W^{1,2}\left( \Omega ;S_0\right)$ is the Soboblev space of square-integrable $\mathbf{Q}$-tensors with square-integrable first derivatives [12], with norm

$$\begin{aligned} \left| \left| \mathbf{Q}\right| \right| _{W^{1,2}} = \left( \int _{\Omega } \left| \mathbf{Q}\right| ^2 + \left| {{\nabla }}\mathbf{Q}\right| ^2~dV \right) ^{1/2}. \end{aligned}$$

In what follows, we identify the degree of a uniaxial $\mathbf{Q}$-tensor in ${\mathcal {A}}$ on the boundary, with the degree of the director field, $\mathbf{n}_b \in W^{1,2}\left( \Omega ; S^2 \right)$ on the boundary, ${\text {deg}}\, (\mathbf{n}_b, \partial \Omega )$, which is well defined because $\mathbf{n}_b$ is smooth. The existence of a global minimizer of $\mathbf{I_{LG}}$ in the admissible space, ${\mathcal {A}}$, is immediate from the direct method in the calculus of variations [12]; the details are omitted for brevity. It follows from standard arguments in elliptic regularity that all global minimizers are smooth and real analytic solutions of the Euler–Lagrange equations associated with $\mathbf{I_{LG}}$ on $\Omega$,

15 $$\begin{aligned} L \Delta \mathbf{Q}= A \mathbf{Q}- B\left( \mathbf{Q}^2 - \left( {\text {tr}}\mathbf{Q}^2\right) \frac{\mathbf {I}}{3}\right) + C \left( {\text {tr}}\mathbf{Q}^2\right) \mathbf{Q}, \end{aligned}$$

where $B\left( {\text {tr}}\mathbf{Q}^2\right) \frac{\mathbf {I}}{3}$ is a Lagrange multiplier accounting for the tracelessness constraint [24].

Define the re-scaled maps, $\bar{\mathbf {Q}}:= \frac{1}{s_+}\sqrt{\frac{3}{2}} \mathbf {Q}$. Let

16 $$\begin{aligned} t:= \frac{27\left| A\right| C}{B^2}, \ h_+=\frac{3+\sqrt{9+8t}}{4}, \ \bar{\mathbf{x}}_i = \frac{\mathbf{x}_i}{D}; \quad \text {and}\quad \bar{L}:= \frac{27C}{ 2 D^2 B^2}L; \end{aligned}$$

where *D* is a characteristic geometrical length scale related to the size of $\Omega$. Then $s_+ = \frac{B}{3C} h_+$ and we work with the re-scaled LdG energy given by

17 $$\begin{aligned} \frac{3 \bar{L}}{2Ls_+^2 D} \mathbf {I}_{LG}\left[ \bar{\mathbf{Q}}\right] = \int _{\Omega } \frac{\bar{L}}{2}\left| \bar{{{\nabla }}} \bar{\mathbf{Q}}\right| ^2 + \frac{t}{8}\left( 1- \left| \bar{\mathbf{Q}}\right| ^2 \right) ^2 + \frac{h_+}{8} \left( 1+3\left| \bar{\mathbf {Q}}\right| ^4-4\sqrt{6}{{\text {tr}}}\bar{\mathbf {Q}}^3\right) ~\bar{dV}. \end{aligned}$$

In (17), we have added a re-scaled constant ($-\min _{\mathbf{Q}\in S_0} f_B\left( \mathbf{Q}\right)$) to the energy density to ensure that the energy density is non-negative.

We point out that $\bar{L}$ can be interpreted as the ratio of the bare biaxial correlation length, $\xi _0 \sim \sqrt{\frac{LC}{B^2}}$, (see [17, 18]) and *D* i.e. $\bar{L} \propto \left( \frac{\xi _0}{D}\right) ^2$. The regime $\xi _u<<\xi _b<<D$ studied in this paper corresponds to $t\rightarrow \infty$ and $\frac{\bar{L}}{h_+}\rightarrow 0$ simultaneously. For clarity and simplicity, **we work with fixed but small values of** $\bar{L}$; there is no loss of generality since all the arguments are valid when $\bar{L}$ varies with *t* but satisfies $\frac{\bar{L}}{h_+}\rightarrow 0$. Further, the bulk potential has two contributions in (17), both of which are nonnegative in view of (8). The first term vanishes for $\bar{\mathbf{Q}} \in \mathbb {S}^4$ and the second term vanishes if and only if $\bar{\mathbf{Q}}$ is uniaxial with unit norm [from (8) again]. The two contributions scale differently as $t \rightarrow \infty$ i.e. the uniaxiality preferred by the second term is a lower order effect compared to the preference for unit norm, as enforced by the first term in the bulk potential. The re-scaled boundary condition is $\bar{\mathbf {Q}}_{b} = \sqrt{\frac{3}{2}}\left( \mathbf{n}_b \otimes \mathbf{n}_b - \frac{\mathbf {I}}{3} \right)$. **In what follows, all statements are to be understood in terms of the re-scaled variables and we drop the bars from the variables for brevity.** We recall the definition of a minimizing limiting harmonic map.

### Definition 1

A (minimizing) limiting harmonic map with respect to the re-scaled Dirichlet condition in (13), is a uniaxial map of the form

18 $$\begin{aligned} \mathbf{Q}^0 = \sqrt{\frac{3}{2}} \left( \mathbf{n}^0 \otimes \mathbf{n}^0 - \frac{\mathbf {I}}{3} \right) , \end{aligned}$$

where $\mathbf{n}^0$ is a minimizer of the Dirichlet energy

19 $$\begin{aligned} I\left[ \mathbf{n}\right] = \int _{\Omega } \left| {{\nabla }}\mathbf{n}\right| ^2~dV \end{aligned}$$

in the admissible space ${\mathcal {A}}_{\mathbf{n}_b} = \left\{ \mathbf{n}\in W^{1,2}\left( \Omega ;\mathbb {S}^2\right) ; \mathbf{n}= \mathbf {n}_b ~on~ \partial \Omega \right\}$ [34].

In particular, $\mathbf{n}^0$ is a harmonic map into $\mathbb {S}^2$, i.e., a solution of the harmonic map equations $\Delta \mathbf{n}+ |{{\nabla }}\mathbf{n}|^2 \mathbf{n}=0$. The singular set of $\mathbf{n}^0$, denoted by $\Sigma =\left\{ \mathbf{x}_1\ldots \mathbf{x}_N \right\} \subset \Omega$, is a finite set of points [34, 35].

We have two main theorems.

### Theorem 1

Let $\Omega \subset \mathbb {R}^3$ be as above. Let $\{t_j\}_{j\in \mathbb {N}}$ with $t_j\overset{j\rightarrow \infty }{\longrightarrow } +\infty$ and let $\{\mathbf {Q}_j\}_{j\in \mathbb {N}}$ be a corresponding global minimizer of the LdG energy in (17), in the admissible space $\bar{{\mathcal {A}}} = \left\{ \mathbf{Q}\in W^{1,2}\left( \Omega ;S_0\right) ; \mathbf{Q}= \sqrt{\frac{3}{2}}\left( \mathbf{n}_b \otimes \mathbf{n}_b - \frac{\mathbf {I}}{3}\right) ~\text {on}~ \partial \Omega \right\} .$ Then (up to a subsequence), we have the following results.

(i) $\{\mathbf {Q}_j\}$ converges to a limiting harmonic map, $\mathbf{Q}^0$ defined in (18), strongly in $W^{1,2}(\Omega ; S_0)$ and uniformly away from $\Sigma$, as $j \rightarrow \infty$.
(ii) Let $\Sigma _{{{\epsilon }}} = \left\{ \mathbf{x}\in \Omega : dist\left( \mathbf{x},\Sigma \right) < {{\epsilon }} \right\} = \bigcup _{\mathbf{x}_i \in \Sigma } B_{{{\epsilon }}}(\mathbf{x}_i)$ where $B_{{{\epsilon }}}(\mathbf{x}_i)$ denotes a ball of radius ${{\epsilon }}$ centered at $\mathbf{x}_i$, and $B_\delta ^j = \left\{ \mathbf{x}\in \Omega : \beta ^2\left( \mathbf{Q}_j(\mathbf{x}) \right) > \delta \right\}$, for a fixed ${{\epsilon }}>0$ and $\delta \in \left( 0, 1 \right)$. Then $B_\delta ^j \subseteq \Sigma _{{{\epsilon }}}$ for *j* large enough.
(iii) $\left| \mathbf{Q}_j \right| \rightarrow 1$ uniformly on $\Omega$ as $j \rightarrow \infty$.
(iv) For each $\mathbf{x}_i \in \Sigma$, we have for *j* large enough,
  20 $$\begin{aligned} \min _{\mathbf{x}\in \overline{B_{{{\epsilon }}}(\mathbf{x}_i)}} \beta ^2\left( \mathbf{Q}_j(\mathbf{x}) \right) =0, \quad \max _{\mathbf{x}\in \overline{B_{{{\epsilon }}}(\mathbf{x}_i)}}\beta ^2\left( \mathbf{Q}_j(\mathbf{x}) \right) = 1, \end{aligned}$$
  and $$\mathcal L^n(\{\mathbf {x} \in \overline{B_{{{\epsilon }}}(\mathbf{x}_i)}: \beta ^2\left( \mathbf {Q}_j(\mathbf {x})\right) =0\})=0$$.
(v) For each $\mathbf{x}_i \in \Sigma$ and $\delta \in \left( 0, 1 \right)$, we have
  21 $$\begin{aligned} {\text {diam}}\left( B_{{\epsilon }}(\mathbf{x}_i) \cap B_\delta ^j \right) \sim t_j^{-1/4} \end{aligned}$$
  for *j* sufficiently large.

An immediate consequence is that global energy minimizers cannot be purely uniaxial, as also stated in [10] where the authors prove that global LdG minimizers must have at least a point of maximal biaxiality.

### Theorem 2

Let $\Omega \subset \mathbb {R}^3$ be as above. Let $\{t_j\}_{j\in \mathbb {N}}$ be such that $t_j \rightarrow \infty$ as $j\rightarrow \infty$. For each $j\in \mathbb {N}$, let $\{\mathbf {Q}_j\}_{j\in \mathbb {N}}$ be a global minimizer of the LdG energy (17) in the restricted class of uniaxial $\mathbf{Q}$-tensors of the form (7). Then $\mathbf {Q}_j$ has non-negative scalar order parameter and $\mathbf {Q}_j$ satisfies the following energy bound

22 $$\begin{aligned} \displaystyle \frac{3\bar{L} }{2Ls_+^2 D} \mathbf {I}_{LG}[\mathbf {Q}_j]\le \frac{3\bar{L}}{2 }\cdot \inf _{\mathbf {n} \in \mathcal A_{\mathbf {n}_b}} I[\mathbf {n}], \end{aligned}$$

with $I[\mathbf {n}]$ and $\mathcal{A}_{\mathbf {n}_b}$ as in (19), for each $j \in \mathbb {N}$. For *j* sufficiently large, $\mathbf {Q}_j$ cannot be a stable critical point of the LdG energy in (17) in the admissible space, $\bar{{\mathcal {A}}} = \left\{ \mathbf{Q}\in W^{1,2}\left( \Omega ;S_0\right) ; \mathbf{Q}= \sqrt{\frac{3}{2}}\left( \mathbf{n}_b \otimes \mathbf{n}_b - \frac{\mathbf {I}}{3}\right) ~\text {on}~ \partial \Omega \right\} .$

Theorem 2 is a consequence of Proposition 2.1 below and Propositions 3 and 8 of [15].

### Proposition 2.1

Let $\Omega \subset \mathbb {R}^3$ be as above. Let $\{t_j\}_{j\in \mathbb {N}}$ be such that $t_j \rightarrow \infty$ as $j\rightarrow \infty$. Let $\mathbf {Q}_j$ be a sequence of uniaxial critical points of the re-scaled LdG energy in (17) with non-negative scalar order parameter and satisfying the energy bound (22) for all $j >0$. Then, passing to a subsequence (still indexed by *j*), the sequence $\{\mathbf {Q}_j\}$ converges uniformly to a (minimizing) limiting harmonic map, $\mathbf{Q}^0$ as $j\rightarrow \infty$, everywhere away from the singular set $\Sigma = \left\{ \mathbf{x}_1 \ldots \mathbf{x}_N\right\}$ of $\mathbf{Q}^0$. We have that

(i) for each $i=1,\ldots , N$, there exists $\{\mathbf {x}_i^{(j)}\}_{j\in \mathbb {N}}$ such that $\mathbf {Q}_{j}(\mathbf {x}_i^{(j)})=\mathbf {0}$ for all $j\in \mathbb {N}$ and $\mathbf {x}_i^{(j)} \overset{j\rightarrow \infty }{\longrightarrow } \mathbf {x}_i \in \Sigma$ and
(ii) given any sequence, $\{\mathbf {x}^{(j)}\}_{j\in \mathbb {N}}\subset \Omega$, such that $\mathbf {Q}_j(\mathbf {x}^{(j)})=\mathbf {0}\ \forall \,j\in \mathbb {N}$, there exists a subsequence $\{j_k\}_{k\in \mathbb {N}}$ and an orthogonal transformation $\mathbf {T}\in \mathcal O(3)$ (which may depend on the subsequence) such that the shifted maps $\displaystyle \left\{ \tilde{\mathbf {x}} \mapsto \mathbf {Q}_{j_k} \left( \mathbf {x}^{(j_k)}+\xi _b \tilde{\mathbf {x}}\right) \right\} _{k\in \mathbb {N}}$ converge to
  23 $$\begin{aligned} \mathbf {H}_{\mathbf {T}}(\tilde{\mathbf {x}}):=\sqrt{\frac{3}{2}} h\left( \left| \tilde{\mathbf {x}}\right| \right) \left( \frac{\mathbf {T} \tilde{\mathbf {x}} \otimes \mathbf {T}\tilde{\mathbf {x}}}{\left| \tilde{\mathbf {x}}\right| ^2} - \frac{\mathbf {I}}{3} \right) , \quad \tilde{\mathbf {x}} \in \mathbb {R}^3, \end{aligned}$$
  in $C^r_{{{\mathrm{loc}}}}(\mathbb {R}^3; S_0)$ for all $r\in \mathbb {N}$, where $h:[0,\infty )\rightarrow \mathbb {R}^+$ is the unique, monotonically increasing solution, with $r=|\tilde{\mathbf {x}}|$, of the boundary-value problem
  24 $$\begin{aligned} \frac{d^2 h}{d r^2} + \frac{2}{r}\frac{dh}{dr} - \frac{6 h}{r^2} = h^3 - h, \qquad h(0)=0, \qquad \lim _{r\rightarrow \infty } h(r)=1. \end{aligned}$$

Proposition 2.1 provides a local description of the structural profile near a set of isotropic points in the uniaxial critical points, $\mathbf{Q}_j$, in terms of the well-known RH solution. The RH solution is a rare example of a semi-explicit critical point of the LdG energy simply given by

25 $$\begin{aligned} \mathbf {H}(\tilde{\mathbf {x}}):=\sqrt{\frac{3}{2}} h\left( \left| \tilde{\mathbf {x}}\right| \right) \left( \frac{ \tilde{\mathbf {x}} \otimes \tilde{\mathbf {x}}}{\left| \tilde{\mathbf {x}}\right| ^2} - \frac{\mathbf {I}}{3} \right) , \quad \tilde{\mathbf {x}} \in \mathbb {R}^3, \end{aligned}$$

where *h* is defined as in (24). The boundary-value problem (24) has been studied in detail, see for example in [19, 23]. The RH solution is locally unstable with respect to biaxial perturbations, as has been demonstrated in [22, 26].

## Proof of the theorems

Recall that the re-scaled LdG energy is given by

26 $$\begin{aligned} \frac{3\bar{L}}{2Ls_+^2 D} \mathbf {I}^j_{LG}\left[ \mathbf{Q}\right] = \int _{\Omega } \frac{\bar{L}}{2}\left| {{\nabla }}\mathbf{Q}\right| ^2 + \bar{L} f(\mathbf {Q}, t_j)~dV, \end{aligned}$$

with

27 $$\begin{aligned} \bar{L} f(\mathbf{Q},t) = \frac{t}{8}\left( 1 - \left| \mathbf{Q}\right| ^2 \right) ^2 + \frac{h_+}{8}\left( 1+ 3\left| \mathbf{Q}\right| ^4 - 4\sqrt{6}{\text {tr}}\mathbf{Q}^3 \right) , \end{aligned}$$

and that for all $t>0$, the potential $f(\mathbf{Q}, t)$ is minimized on the set

28 $$\begin{aligned} \mathbf{Q}_{\min } = \left\{ \sqrt{\frac{3}{2}}\left( \mathbf{n}\otimes \mathbf{n}- \frac{\mathbf {I}}{3} \right) : \mathbf {n} \in \mathbb {S}^2 \right\} . \end{aligned}$$

Denote the LdG energy density by

29 $$\begin{aligned} e(\mathbf{Q},t) =\frac{1}{2}\left| {{\nabla }}\mathbf{Q}\right| ^2 + f(\mathbf{Q}, t). \end{aligned}$$

In Theorem 1 we consider global minimizers $\mathbf{Q}_j$ of (26) in the admissible space, $\bar{{\mathcal {A}}} = \left\{ \mathbf{Q}\in W^{1,2}\left( \Omega ;S_0\right) ; \mathbf{Q}= \sqrt{\frac{3}{2}}\left( \mathbf{n}_b \otimes \mathbf{n}_b - \frac{\mathbf {I}}{3}\right) ~\text {on}~ \partial \Omega \right\}$, for each $t_j>0$, the existence of which is guaranteed by the direct method of the calculus of variations. Standard elliptic regularity arguments (presented in [24, Prop. 13]) show that each minimizer $\mathbf {Q}_j$ is a real analytic solution of the Euler-Lagrange equations

30 $$\begin{aligned} \triangle \mathbf{Q}_{ij} = \Gamma _{ij}, \end{aligned}$$

where

$$\begin{aligned} \bar{L}\Gamma _{ij} = \frac{t}{2}\mathbf{Q}_{ij}\left( \left| \mathbf{Q}\right| ^2 - 1 \right) + \frac{3 h_+}{2}\left[ \left| \mathbf{Q}\right| ^2 \mathbf{Q}_{ij} - \sqrt{6}\mathbf{Q}_{ip}\mathbf{Q}_{pj} + \sqrt{6}\left| \mathbf{Q}\right| ^2\delta _{ij}/3 \right] . \end{aligned}$$

Theorem 2 is proved by assuming that a sequence $\{\mathbf {Q}_j\}_{j\in \mathbb {N}}$ of minimizers in the restricted class of uniaxial $\mathbf {Q}$-tensors is composed of stable critical points of the LdG energy and then reaching a contradiction. In both cases, we consider classical solutions of (30) that satisfy the energy bound (22) (this follows from the fact that any minimizing limiting harmonic map $\mathbf{Q}^0$ belongs to $\bar{\mathcal {A}}$, so it can be used as a trial function). As done in [10, 15], the arguments in [24, Lemmas 2 and 3; Props. 3, 4, and 6] can be adapted to prove the following preliminary results.

### Proposition 3.1

Let $t_j\rightarrow +\infty$ and, for each $j\in \mathbb {N}$, let $\mathbf {Q}_j\in \bar{{\mathcal {A}}}$ be a classical solution of the corresponding equations (30), satisfying the energy bound (22). Then, passing to a subsequence,

(i) $\{\mathbf {Q}_j\}_{j\in \mathbb {N}}$ converges strongly to a (minimizing) limiting harmonic map $\mathbf {Q}^0$ in $W^{1,2}(\Omega ; S_0)$,
(ii) $\Vert \mathbf{Q}_j \Vert _{L^{\infty }} \le 1$ and $\Vert {{\nabla }}\mathbf{Q}_j \Vert _{L^{\infty }} \le C \sqrt{\frac{t_j}{\bar{L}}}$ for some *C* independent of *j*,
(iii) $\displaystyle \frac{1}{r}\int _{B(\mathbf {x},r)} e(\mathbf {Q}_j, t_j)~dV \le \frac{1}{R}\int _{B(\mathbf {x}, R)} e(\mathbf {Q}_j, t_j)~dV$ for all $\mathbf {x} \in \Omega$ and $r\le R$ so that $B(\mathbf {x}, R)\subset \Omega$,
(iv) for any compact $K\subset \overline{\Omega }\setminus \Sigma$, where $\Sigma$ denotes the singular set of $\mathbf{Q}^0$,
  31 $$\begin{aligned} \frac{1}{8}\left( 1 - \left| \mathbf{Q}_j\right| ^2 \right) ^2 + \frac{h_+}{8 t}\left( 1 + 3\left| \mathbf{Q}_j\right| ^4 - 4 \sqrt{6}{{\mathrm{tr}}}\mathbf{Q}_j^3\right) \rightarrow 0 \end{aligned}$$
  uniformly in *K*.

However, this only ensures that $|\mathbf{Q}_j| \rightarrow 1$ uniformly as $j \rightarrow \infty$, away from $\Sigma$. By contrast, in [24], the authors could prove that for a fixed $t>0$, the global minimizers uniformly approach $\mathbf{Q}_{\min }$, everywhere away from $\Sigma$, as $L \rightarrow 0$ in (1). The analysis in [24] would carry over to the $\bar{L} \rightarrow 0$ limit i.e. when the domain is large compared to a characteristic material-dependent correlation length. We want to prove the following result.

### Proposition 3.2

Under the hypotheses of Proposition 3.1, $\{\mathbf {Q}_j\}_{j\in \mathbb {N}}$ converges uniformly to $\mathbf {Q}^0$ away from $\Sigma$, as $t_j\rightarrow \infty$.

The key step is to prove a Bochner inequality of the form

### Lemma 3.3

There exist ${\epsilon }_1>0$ and a constant $C>0$ independent of *t* such that if $\mathbf {Q}\in C^3(\Omega ; S_0)$ is a solution of (30), then

32 $$\begin{aligned} -\triangle e\left( \mathbf{Q}, t \right) \left( \mathbf{x}\right) \le C e^2\left( \mathbf{Q}, t \right) \left( \mathbf{x}\right) \end{aligned}$$

for all $\mathbf {x}\in \Omega$ satisfying

33 $$\begin{aligned} 1- {\epsilon }_1 \le \left| \mathbf{Q}(\mathbf {x})\right| \le 1. \end{aligned}$$

This inequality is proven in [24, Lemmas 5–7] in the case when $\mathbf{Q}_j$ is close to the manifold $\mathbf{Q}_{\min }$, defined in (28), which does not necessarily hold in our case as explained below.

### Proof of Lemma 3.3

The quantity $\left| \mathbf{Q}\right| ^3 - \sqrt{6} {\text {tr}}\mathbf{Q}^3$ plays an important role and we note the elementary inequality

34 $$\begin{aligned} 0 \le \left( \left| \mathbf{Q}\right| ^3 - \sqrt{6} {\text {tr}}\mathbf{Q}^3 \right) \le \frac{\left( 3 - {{\mathrm{sgn}}}{\text {tr}}\mathbf{Q}^3 \right) }{2} \left| \mathbf{Q}\right| ^3. \end{aligned}$$

This quantity controls the biaxiality parameter as shown below:

35 $$\begin{aligned} \beta ^2(\mathbf {Q})&= \frac{\left| \mathbf {Q}\right| ^3-\sqrt{6}{{\mathrm{tr}}}\mathbf {Q}^3}{\left| \mathbf {Q}\right| ^3} \frac{\left| \mathbf {Q}\right| ^3+\sqrt{6}{{\mathrm{tr}}}\mathbf {Q}^3}{\left| \mathbf {Q}\right| ^3}\end{aligned}$$

36 $$\begin{aligned}&\le \frac{\left| \mathbf {Q}\right| ^3-\sqrt{6}{{\mathrm{tr}}}\mathbf {Q}^3}{(1-{\epsilon }_1)^3} \cdot \frac{\left| \mathbf {Q}\right| ^3 + \left| \mathbf {Q}\right| ^3}{\left| \mathbf {Q}\right| ^3} \le 2 \frac{\left| \mathbf {Q}\right| ^3-\sqrt{6}{{\mathrm{tr}}}\mathbf {Q}^3}{(1-{\epsilon }_1)^3} \end{aligned}$$

since $1 - |\mathbf{Q}(\mathbf{x})|\le \epsilon _1$ from (33) by assumption.

As in [24], we use the Euler–Lagrange equations (30) to derive the following inequality:

37 $$\begin{aligned} -\triangle e\left( \mathbf{Q}, t \right) +\left| \Gamma \right| ^2 \le - 2\frac{\partial ^2 f}{\partial \mathbf{Q}_{ij}\partial \mathbf{Q}_{pq}}\mathbf{Q}_{ij,k} \mathbf{Q}_{pq,k}. \end{aligned}$$

Moreover, we have

38 $$\begin{aligned} \bar{L}^2\left| \Gamma \right| ^2= & {} \frac{t^2}{4}\left| \mathbf{Q}\right| ^2\left( 1 - \left| \mathbf{Q}\right| ^2 \right) ^2 + \frac{9 h_+^2}{4}\left( 1 - \left| \mathbf{Q}\right| \right) ^2 \left| \mathbf{Q}\right| ^4 \nonumber \\&+\, \frac{3 h_+ t}{2}\left( 1 - \left| \mathbf{Q}\right| ^2\right) \left( \left| \mathbf{Q}\right| ^3 - \left| \mathbf{Q}\right| ^4\right) +\frac{3 h_+ t}{2}\left( \left| \mathbf{Q}\right| ^2 - 1\right) \left( \left| \mathbf{Q}\right| ^3 - \sqrt{6}{\text {tr}}\mathbf{Q}^3\right) \nonumber \\&+\,\frac{9h_+^2}{2}\left( \left| \mathbf{Q}\right| ^3 - \sqrt{6}{\text {tr}}\mathbf{Q}^3\right) \left| \mathbf{Q}\right| ^2 \end{aligned}$$

and

39 $$\begin{aligned}&- \bar{L}\frac{\partial ^2 f}{\partial \mathbf{Q}_{ij}\partial \mathbf{Q}_{pq}}\mathbf{Q}_{ij,k} \mathbf{Q}_{pq,k} = \frac{t}{2}\left| {{\nabla }}\mathbf{Q}\right| ^2\left( 1 - \left| \mathbf{Q}\right| ^2\right) -t(\mathbf{Q}\cdot {{\nabla }}\mathbf{Q})^2\nonumber \\&\quad -\, 3 h_+\left( \mathbf{Q}\cdot {{\nabla }}\mathbf{Q}\right) ^2 - \frac{3 h_+}{2}\left| \mathbf{Q}\right| ^2 \left| {{\nabla }}\mathbf{Q}\right| ^2 + 3\sqrt{6} h_+ \mathbf{Q}_{\beta j}\mathbf{Q}_{\alpha j,k}\mathbf{Q}_{\alpha \beta ,k}. \end{aligned}$$

We consider three separate cases according to the sign of ${\text {tr}}\mathbf{Q}^3$ and the magnitude of $\left| \mathbf{Q}\right| ^3 - \sqrt{6}{\text {tr}}\mathbf{Q}^3$.

*Case I* $0\le \left| \mathbf{Q}\right| ^3 - \sqrt{6}{\text {tr}}\mathbf{Q}^3 \le {\epsilon }_1$. This, when combined with (33), implies that ${\text {tr}}\mathbf{Q}^3>0$ and that the biaxiality parameter, $\beta ^2(\mathbf{Q}) \le \frac{2{\epsilon }_1}{(1 - {\epsilon }_1)^3}$, so that $\mathbf{Q}$ is approximately uniaxial with unit norm for ${\epsilon }_1$ sufficiently small. Equivalently, $\left| \mathbf{Q}- \sqrt{\frac{3}{2}} \left( \mathbf{n}\otimes \mathbf{n}- \frac{\mathbf {I}}{3} \right) \right| \le {\epsilon }_0$ for some $\mathbf{n}\in S^2$ (where ${\epsilon }_0$ depends on ${\epsilon }_1$). In this case, we can repeat all the arguments in [24, Lemmas 5–7]; we state the key steps for completeness.

We denote the eigenvectors of $\mathbf{Q}$ by $\mathbf{n}_1, \mathbf{n}_2, \mathbf{n}_3$ respectively and let ${\lambda }_3>0$ and ${\lambda }_1, {\lambda }_2$ denote the corresponding eigenvalues. Define

$$\begin{aligned} \mathbf{Q}^* = \sqrt{\frac{2}{3}}\mathbf{n}_3 \otimes \mathbf{n}_3 - \sqrt{\frac{1}{6}}\left( \mathbf{n}_1 \otimes \mathbf{n}_1 + \mathbf{n}_2\otimes \mathbf{n}_2 \right) . \end{aligned}$$

From the inequality $\left| \mathbf{Q}- \sqrt{\frac{3}{2}}\left( \mathbf{n}\otimes \mathbf{n}- \frac{\mathbf {I}}{3} \right) \right| \le {\epsilon }_0$ with $\mathbf{n}=\mathbf{n}_3$, we necessarily have that

$$\begin{aligned} \left( {\lambda }_1 + \sqrt{\frac{1}{6}}\right) ^2 + \left( {\lambda }_2 + \sqrt{\frac{1}{6}}\right) ^2 + \left( {\lambda }_3 - \sqrt{\frac{2}{3}}\right) ^2 \le {\epsilon }_0^2. \end{aligned}$$

One can repeat the arguments of [24, Lemma 5] verbatim to show that there exists a positive constant ${\epsilon }_0>0$ such that:

40 $$\begin{aligned} \frac{t}{C}f(\mathbf{Q}, t) \le \left| \Gamma \right| ^2(\mathbf{Q}, t) \le C t f(\mathbf{Q}, t) \end{aligned}$$

for all $\mathbf{Q}\in S_0$ with $\left| \mathbf{Q}- \sqrt{\frac{3}{2}} \left( \mathbf{n}\otimes \mathbf{n}- \frac{\mathbf {I}}{3} \right) \right| \le {\epsilon }_0$ where $\mathbf{n}\in \mathbb {S}^2$, the positive constant *C* being independent of *t*.

The proof of the Bochner inequality now follows from the chain of inequalities:

41 $$\begin{aligned}&-\triangle e(\mathbf{Q}, t) + \left| \Gamma \right| ^2 \le - 2\frac{\partial ^2 f}{\partial \mathbf{Q}_{ij}\partial \mathbf{Q}_{pq}}\mathbf{Q}_{ij,k} \mathbf{Q}_{pq,k}\nonumber \\&\le \delta \sum _{i,j,m=1}^{3} \left( \frac{\partial ^3 f}{\partial \mathbf{Q}_{ij}\mathbf{Q}_{pq}\partial \mathbf{Q}_{mn} } \left( \mathbf{Q}^*\right) \right) ^2 \left( \mathbf{Q}- \mathbf{Q}^*\right) ^2\end{aligned}$$

42 $$\begin{aligned}&\quad +\,\delta \sum _{i,j,m=1}^{3} \left( \mathcal {R}^{ijmn} \right) ^2 \left( \mathbf{Q}, \mathbf{Q}^* \right) + \frac{1}{\delta }\left| {{\nabla }}\mathbf{Q}\right| ^{4}\nonumber \\&\le C_1 \delta t^2 \left| \mathbf{Q}- \mathbf{Q}^* \right| ^2 + \frac{1}{\delta }\left| {{\nabla }}\mathbf{Q}\right| ^{4}\nonumber \\&\le C_2 \delta t f(\mathbf{Q}, t) + \frac{1}{\delta }|{{\nabla }}\mathbf{Q}|^4 . \end{aligned}$$

In the above, we have followed Equations $(66)--(68)$ of [24] to deduce that

$$\begin{aligned} t^2 \left| \mathbf{Q}- \mathbf{Q}^* \right| ^2 \le C^{*} t f\left( \mathbf{Q}, t \right) \end{aligned}$$

for some positive constant $C^*$ independent of *t*, for $\mathbf{Q}$ sufficiently close to $\mathbf{Q}_{\min }$, as is the case here. In Eq. (41) above, we have carried out a Taylor series expansion of the right-hand side of (37) about $\mathbf{Q}^*$, $\left( \mathcal {R}^{ijmn} \right)$ is the remainder term in the Taylor series expansion which is well-controlled and the constants $C_1$ and $C_2$ are independent of *t* but dependent on $\bar{L}$ (which does not matter since $\bar{L}$ is fixed). For $\delta$ sufficiently small, we can absorb the $C_2 \delta t f(\mathbf{Q}, t)$-term on the right by the $\left| \Gamma \right| ^2(\mathbf{Q})$-contribution on the left so that

$$\begin{aligned} \left| \Gamma \right| ^2(\mathbf{Q}_j) - C_2 \delta t f(\mathbf{Q}, t) \ge 0 \end{aligned}$$

for $\delta$ sufficiently small [from (40)], yielding the Bochner inequality

$$\begin{aligned} -\triangle e(\mathbf{Q}, t) \le \frac{1}{\delta }\left| {{\nabla }}\mathbf{Q}\right| ^4 \end{aligned}$$

for $\delta >0$ independent of *t*, as required.

*Case II* ${\text {tr}}\mathbf{Q}^3>0$ and ${\epsilon }_1 <\left| \mathbf{Q}\right| ^3 - \sqrt{6}{\text {tr}}\mathbf{Q}^3 \le 1$. The key difference between Case I and Cases II and III is that $\beta ^2(\mathbf{Q})$ need not be small for Cases II and III i.e. $\mathbf{Q}$ need not be close to the manifold, $\mathbf{Q}_{\min }$, rendering these cases outside the scope of the results in [24].

We refer to the relations (37)–(39) and use the Cauchy–Schwarz inequality in (38) to see that

$$\begin{aligned} \frac{3 h_+ t}{2}\left( \left| \mathbf{Q}\right| ^2 - 1\right) \left( \left| \mathbf{Q}\right| ^3 - \sqrt{6}{\text {tr}}\mathbf{Q}^3\right) \ge - \delta t^2\left( \left| \mathbf{Q}\right| ^2 - 1\right) ^2 - \frac{9 h_+^2}{16}\frac{1}{\delta }\left( \left| \mathbf{Q}\right| ^3 - \sqrt{6}{\text {tr}}\mathbf{Q}^3\right) ^2. \end{aligned}$$

For $\frac{3}{16}< \delta < \frac{1-2{\epsilon }_1}{4}$ and ${\epsilon }_1$ chosen as above, we have

43 $$\begin{aligned} \bar{L}^2\left| \Gamma \right| ^2\ge & {} \alpha t^2 \left| \mathbf{Q}\right| ^2\left( 1 - \left| \mathbf{Q}\right| \right) ^2 + \frac{9 h_+^2}{4}\left( 1 - \left| \mathbf{Q}\right| \right) ^2\left| \mathbf{Q}\right| ^4 \nonumber \\&+\,\frac{3 h_+ t}{2} \left| \mathbf{Q}\right| ^3 \left( 1 - \left| \mathbf{Q}\right| \right) ^2 \left( 1 + \left| \mathbf{Q}\right| \right) + \eta h_+^2\left( \left| \mathbf{Q}\right| ^3 - \sqrt{6}{\text {tr}}\mathbf{Q}^3\right) \end{aligned}$$

for positive constants $\alpha , \eta$ independent of *t* and $\bar{L}$ is fixed for our purposes. Finally, we appeal to (39) to obtain

44 $$\begin{aligned}&- \bar{L}\frac{\partial ^2 f}{\partial \mathbf{Q}_{ij}\partial \mathbf{Q}_{pq}}\mathbf{Q}_{ij,k} \mathbf{Q}_{pq,k}\le \delta _1 \frac{t^2}{4}\left( 1 - \left| \mathbf{Q}\right| ^2\right) ^2 \nonumber \\&\quad +\,\delta _2 h_+^2 \left| \mathbf{Q}\right| ^2 + \frac{1}{\delta _5(\delta _1, \delta _2)}\left| {{\nabla }}\mathbf{Q}\right| ^4. \end{aligned}$$

For $\delta _2$ sufficiently small, we can absorb the $h_+^2|\mathbf{Q}|^2$ term in (44) by the $\eta h_+^2(|\mathbf{Q}|^3 - \sqrt{6}{\text {tr}}\mathbf{Q}^3)$ term in (43). Choosing $\delta _1,\delta _2$ small enough (and independent of *t*), recalling (37), the lower bound (43) and the upper bound (44), we have

45 $$\begin{aligned} -\triangle e\left( \mathbf{Q}, t \right) \le \frac{1}{\delta _5} \left| {{\nabla }}\mathbf{Q}\right| ^4 \end{aligned}$$

which is precisely the Bochner inequality.

*Case III* ${\text {tr}} \mathbf{Q}^3 \le 0$ so that $\left( 1- {\epsilon }_1\right) ^3 \le \left| \mathbf{Q}\right| ^3 - \sqrt{6}{\text {tr}}\mathbf{Q}^3 \le 2 |\mathbf{Q}|^3$.

A large part of the computations for Case II carry over to Case III. In particular, (44) is unchanged and it remains to note that for ${\text {tr}}\mathbf{Q}^3 < 0$, the bulk potential $\bar{L}f(\mathbf{Q},t) \ge \frac{t}{8}\left( 1 - |\mathbf{Q}|^2 \right) ^2 + \frac{h_+}{8}$. In particular,

46 $$\begin{aligned} \frac{h_+^2}{64{\bar{L}}^2} \le e^2 (\mathbf{Q},t). \end{aligned}$$

Define $\sigma$ and $\gamma$ to be

47 $$\begin{aligned} 1 - \left| \mathbf{Q}\right| ^2= & {} \sigma \frac{h_+}{t} \nonumber \\ \gamma= & {} \left| \mathbf{Q}\right| ^3 - \sqrt{6} {\text {tr}}\mathbf{Q}^3 \end{aligned}$$

where ${\text {tr}}\mathbf{Q}^3 \le 0$ by assumption. The second, third and fifth terms in (38) are positive, hence

48 $$\begin{aligned} {\bar{L}}^2 \left| \Gamma \right| ^2\ge & {} \frac{\left| \mathbf {Q}\right| ^2}{4} \sigma ^2 h_+^2- \frac{3h_+^2}{2}\sigma \gamma \nonumber \\\ge & {} \frac{h_+^2}{8} \left( \sigma ^2-12\sigma \gamma \right) \ge \frac{h_+^2}{8} \left( (\sigma - 6\gamma )^2 - 36 \gamma ^2\right) \ge -\frac{9h_+^2}{2} \gamma ^2. \end{aligned}$$

Since $\gamma = |\mathbf {Q}|^3 - \sqrt{6}{{\mathrm{tr}}}\mathbf {Q}^3 \le 2$, we get ${\bar{L}}^2 |\Gamma |^2 \ge -18h_+^2$ and therefore, the Bochner inequality (32) then follows from (37), (44), (48), (46). $\square$

The uniform convergence result in Proposition 3.1 (also see the maximum principle in [22]) ensures that $|\mathbf {Q}_j|\rightarrow 1$ away from the singularities of $\mathbf {Q}^0$ and hence, (33) is satisfied for all $t_j$ sufficiently large. Thus, Bochner’s inequality holds away from $\Sigma$ for large *t*, allowing us to deduce the following $\epsilon$-regularity property, exactly as in [24, Lemma 7]:

### Lemma 3.4

Let $K\subset \Omega$ be a compact subset that does not contain any singularity of $\mathbf{Q}^0$. Then there exist $j_0$ and constants $C_1, C_2>0$ (independent of *j*) so that if for $\mathbf {a} \in K$ and $0< r <{{\mathrm{dist}}}\left( \mathbf {a}, \partial K \right)$, we have

49 $$\begin{aligned} \frac{1}{r} \int _{B(\mathbf {a},r)}e\left( \mathbf{Q}_j, t_j \right) ~dV \le C_1, \end{aligned}$$

then

50 $$\begin{aligned} r^2 \sup _{B(\mathbf {a}, r/2)} e\left( \mathbf{Q}_j, t_j \right) \le C_2 \end{aligned}$$

for all $$j\ge j_0$$.

### Proof of Proposition 3.2

The normalized energy, $\frac{1}{r} \int _{B(a,r)}e\left( \mathbf{Q}_j, t_j \right) ~dV$, can be controlled away from $\Sigma$, by simply (i) using the strong convergence of the sequence, $\left\{ \mathbf{Q}_j \right\}$ to $\mathbf{Q}^0$ as $j \rightarrow \infty$ in $W^{1,2}$ and (ii) the fact that $|{{\nabla }}\mathbf{Q}^0|$ is bounded away from $\Sigma$, independently of $t_j$. Thus, the uniform convergence, $\mathbf{Q}_j \rightarrow \mathbf{Q}^0$, away from $\Sigma$ as $j \rightarrow \infty$, follows immediately from Lemma 3.4, combining (49) and (50) and Ascoli–Arzelá Theorem. $\square$

We are almost ready to prove the main theorems. It only remains to state a standard result from homotopy theory and to recall that the Landau–de Gennes energy functional has a Ginzburg–Landau like structure by blowing-up at scale $t^{-1/2}$ and working in the $t\rightarrow \infty$ limit.

### Lemma 3.5

Let $\mathbf {Q}^*(\mathbf {x}):= \displaystyle \sqrt{\frac{3}{2}} \left( \mathbf {n}^*(\mathbf {x}) \otimes \mathbf {n}^*(\mathbf {x}) -\frac{\mathbf {I}}{3}\right)$ for some $\mathbf {n}^*\in C(\partial B; \mathbb {S}^2)$, where *B* is a ball $B(\mathbf {a}, {\epsilon })\subset \mathbb {R}^3$. Suppose that $\mathbf {Q}^*$ is homotopic in $C(\partial B; \mathbf {Q}_{\min })$ [see (28)] to $\mathbf {Q}|_{\partial B}$ for some $\mathbf {Q}\in C(\overline{B}; \mathbf {Q}_{\min })$. Then $\deg \mathbf {n}^*=0$.

### Proof

Since $\mathbf {Q}|_{\partial B}$ has a continuous $\mathbf {Q}_{\min }$-valued extension inside $\overline{B}$, it is homotopic in $C(\partial B; \mathbf {Q}_{\min })$ to the constant tensor $\mathbf {Q}(\mathbf {a})$. Hence, combining the two homotopies, we deduce that $\mathbf{Q}^*$ is homotopic to a constant in $C(\partial B; \mathbf {Q}_{\min })$.

Since $\partial B$ is simply-connected and $\mathbb {S}^2$ is a universal cover of $\mathbf {Q}_{\min }\cong \mathbb {R}P^2$, the latter homotopy lifts to $\mathbb {S}^2$, implying that $\mathbf {n}^*$ is homotopic to a constant in $C(\partial B;\mathbb {S}^2)$ and hence, $\deg \mathbf {n}^*=0$, as needed. $\square$

### Lemma 3.6

Let $t_j\rightarrow +\infty$ and, for each $j\in \mathbb {N}$, let $\mathbf {Q}_j\in \bar{{\mathcal {A}}}$ be a classical solution of (30). Suppose that $\mathbf {Q}_j$ converges strongly in $W^{1,2}$ to a minimizing limiting harmonic map $\mathbf {Q}^0$. Let $\mathbf {x}_j^*$ be a sequence of points converging to some $\mathbf {x}^*$ in the singular set $\Sigma$ of $\mathbf {Q}^0$. Then (up to a subsequence) the rescaled maps

51 $$\begin{aligned} \xi _{j}:= {\sqrt{\frac{\bar{L}}{t_{j}}}}, \qquad {\tilde{\mathbf {x}}}:= \frac{\mathbf {x}-\mathbf {x}_{j}^{*}}{\xi _{j}}, \qquad {\tilde{\mathbf {Q}}}_j({\tilde{\mathbf {x}}}):= \mathbf {Q}_j\left( \mathbf {x}_j^{*} + \xi _{j} \tilde{\mathbf {x}}\right) \end{aligned}$$

converge in $C^k_{{{\mathrm{loc}}}}(\mathbb {R}^3;S_0)$ for all $k\in \mathbb {N}$ to a smooth solution of the Ginzburg–Landau equations $\Delta {\tilde{\mathbf {Q}}}= (|{\tilde{\mathbf {Q}}}|^2-1){\tilde{\mathbf {Q}}}$, in $\mathbb {R}^3$, which satisfies the energy bound

52 $$\begin{aligned} \frac{1}{R}\int _{\left| {\tilde{\mathbf {x}}}\right| <R} \frac{1}{2}\left| \nabla {{\tilde{\mathbf {Q}}}}^\infty ({\tilde{\mathbf {x}}})\right| ^2 + \frac{\left( 1-\left| {{\tilde{\mathbf {Q}}}}^\infty \right| ^2\right) ^2}{8}~\, {\mathrm {d}}V \le 12\pi \quad \forall \,R>0. \end{aligned}$$

### Proof

The proof follows from the celebrated energy quantization result for minimizing harmonic maps at singular points, established in [6]:

53 $$\begin{aligned} \lim _{r\rightarrow 0} \frac{1}{r} \int _{B\left( \mathbf {x}^*, r\right) } \frac{1}{2}\left| \nabla \mathbf {n}^0\right| ^2 \, {\mathrm {d}}V = 4\pi , \quad i=1,\ldots , N. \end{aligned}$$

We begin by noting that $|{{\nabla }}\mathbf{Q}^0|^2 = 3|{{\nabla }}\mathbf{n}^0|^2$, therefore

54 $$\begin{aligned} \frac{1}{r}\int _{B(\mathbf {x}^*, r)} \frac{1}{2}\left| \nabla \mathbf {Q}_j\right| ^2 + f\left( \mathbf {Q}_j,t_j\right) ~\, {\mathrm {d}}V \ \overset{j\rightarrow \infty }{\longrightarrow }\ \frac{3}{r}\int _{B(\mathbf {x}^*, r)} \frac{1}{2}\left| \nabla \mathbf {n}^0\right| ^2~\, {\mathrm {d}}V \end{aligned}$$

for every small $r>0$.

By the monotonicity formula, Proposition 3.1 (iii), for every fixed $R>0$, every small $r>|\mathbf {x}_j^{*}-\mathbf {x}^*| + \xi _j R$, and every *j* sufficiently large, we have that

55 $$\begin{aligned}&\frac{1}{R}\int _{\left| {\tilde{\mathbf {x}}}\right| <R} \frac{1}{2}\left| \nabla {\tilde{\mathbf {Q}}}_j({\tilde{\mathbf {x}}})\right| ^2 + \frac{\left( 1-\left| {\tilde{\mathbf {Q}}}_j\right| ^2\right) ^2}{8} \, {\mathrm {d}}V\end{aligned}$$

56 $$\begin{aligned}&\le \frac{1}{\xi _j R} \int _{\left| \mathbf {x}-\mathbf {x}_j^{*}\right| <\xi _j R} \frac{1}{2}\left| \nabla \mathbf {Q}_j(\mathbf {x})\right| ^2+f (\mathbf {Q}_j(\mathbf {x}), t_j)~\, {\mathrm {d}}V\end{aligned}$$

57 $$\begin{aligned}&\le \frac{1}{r-\left| \mathbf {x}_j^{*}-\mathbf {x}^*\right| } \int _{B\left( \mathbf {x}_j^{*}, r-\left| \mathbf {x}_j^{*}-\mathbf {x}^*\right| \right) } \frac{1}{2}\left| \nabla \mathbf {Q}_j(\mathbf {x})\right| ^2+f \left( \mathbf {Q}_j(\mathbf {x}), t_j\right) ~\, {\mathrm {d}}V\end{aligned}$$

58 $$\begin{aligned}&\le \frac{r}{r-\left| \mathbf {x}_j^{*}-\mathbf {x}^*\right| }\cdot \frac{1}{r}\int _{B(\mathbf {x}^*, r)} \frac{1}{2}\left| \nabla \mathbf {Q}_j(\mathbf {x})\right| ^2+f (\mathbf {Q}_j(\mathbf {x}), t_j)~\, {\mathrm {d}}V \end{aligned}$$

(we have used the inequality $\frac{t}{8\bar{L}}(1-|{\tilde{\mathbf {Q}}}_j |^2)^2 \le f({\tilde{\mathbf {Q}}}_j, t_j)$ above). This combined with (54) and (53) yields the following inequality

59 $$\begin{aligned} \begin{aligned}&\limsup _{j\rightarrow \infty } \frac{1}{R}\int _{\left| {\tilde{\mathbf {x}}}\right| <R} \frac{1}{2}\left| \nabla {\tilde{\mathbf {Q}}}_j({\tilde{\mathbf {x}}})\right| ^2 + \frac{\left( 1-\left| {\tilde{\mathbf {Q}}}_j\right| ^2\right) ^2}{8}~\, {\mathrm {d}}V\\&\quad \le 3\left( \limsup _{r\rightarrow 0^+} \frac{1}{r}\int _{B(\mathbf {x}^*, r)} \frac{1}{2}\left| \nabla \mathbf {n}^0\right| ^2~\, {\mathrm {d}}V\right) \le 12\pi \end{aligned} \end{aligned}$$

for every $$R>0$$.

Using the energy bound above, we can extract a diagonal subsequence, converging weakly in $W^{1,2}_{{{\mathrm{loc}}}}\cap L^4_{{{\mathrm{loc}}}}(\mathbb {R}^3; S_0)$, to a limit map ${\tilde{\mathbf {Q}}}^\infty$ satisfying the energy bound (52). One can check that ${\tilde{\mathbf {Q}}}^\infty$ solves the weak form of the Ginzburg–Landau equations in $\mathbb {R}^3$ (write the weak form of the partial differential equations for ${\tilde{\mathbf {Q}}}_{j_k}$ and pass to the limit when $k\rightarrow \infty$). Standard arguments in elliptic regularity then show that ${\tilde{\mathbf {Q}}}^\infty$ is a classical solution of the Ginzburg–Landau equations and that the diagonal subsequence converges in $\bigcap _{k\in \mathbb {N}} C^{k}_{{{\mathrm{loc}}}}$ to ${\tilde{\mathbf {Q}}}^\infty$. $\square$

### Proof of Theorem 1

(i) It follows from Propositions 3.1 and 3.2.

(ii) This is an immediate consequence of the uniform convergence, $\mathbf{Q}_j \rightarrow \mathbf{Q}^0$ as $j \rightarrow \infty$, away from the singular set, $\Sigma =\left\{ \mathbf{x}_1 \ldots \mathbf{x}_N \right\}$ of $\mathbf{Q}^0$. $\mathbf{Q}^0$ is purely uniaxial by definition i.e. $\beta ^2\left( \mathbf{Q}^0 \right) = 0$ [see (8) for the definition of the biaxiality parameter, $\beta ^2(\mathbf{Q})$]. The map $\mathbf{Q}\mapsto \beta ^2\left( \mathbf{Q}\right)$ is continuous for $\mathbf{Q}\ne 0$ and the conclusion, $B_\delta ^j \subseteq \Sigma _{{{\epsilon }}}$, follows for any fixed ${{\epsilon }}$, provided *j* is large enough.

(iii) This can be proven as in [10], where the authors prove that $|\mathbf{Q}_j(\mathbf{x})|>0$ on $\Omega$, for *j* large enough. We argue by contradiction and we assume that there exist points $\mathbf{x}_j^* \in \Omega$ such that $\left| \mathbf{Q}_j\left( \mathbf{x}_j^*\right) \right| \le 1 - \eta$ (for some $\eta >0$ independent of *j*), for all *j* in the sequence.

In view of part (i), we may assume $\mathbf{x}_j^* \rightarrow \mathbf{x}^*$ for some $\mathbf{x}^* \in \Sigma$ and repeat the arguments in Lemmas 3.1 and 4.1 of [10] i.e. perform a blow-up analysis of the re-scaled maps, $\mathbf{Q}^*_j(\mathbf{x}) = \mathbf{Q}_j\left( \mathbf{x}_j^* + \frac{\mathbf{x}}{\sqrt{t_j}} \right)$. By Lemma 3.6 and [10, Lemma 3.1], the rescaled minimizers converge locally smoothly to a minimizer, $\mathbf{Q}_\infty \in C^2(\mathbb {R}^3; S_0)$, of the Ginzburg–Landau energy,

60 $$\begin{aligned} GL(\mathbf{Q}; A)=\int _A \left| \nabla \mathbf{Q}\right| ^2 +\frac{1}{4} \left( 1-\left| \mathbf{Q}\right| ^2\right) ^2 dV \end{aligned}$$

(on open sets with compact closure $\subset \mathbb {R}^3$ with respect to its own boundary conditions) with the energy growth $GL(\mathbf{Q};B_R(0)) = \mathcal {O} ( R)$ as $R \rightarrow \infty$. In addition we have $|\mathbf{Q}_\infty (0)| \le 1-\eta$ because of the normalization. We can then use the same blow-down analysis as in [10] to show that $\mathbf{Q}_R(\mathbf{x}) = \mathbf{Q}_\infty \left( R \mathbf{x}\right)$ converges strongly in $W^{1,2}_{loc}$ to a $\mathbb {S}^4$-valued minimizing harmonic map, labelled by $\hat{\mathbf{Q}}_\infty$, as $R\rightarrow \infty$. Indeed, one can use the well-known Luckhaus interpolation Lemma as in [29], Proposition 4.4, still for a sequence of functionals converging to the Dirichlet integral for maps into a manifold, showing that minimality persist in the limit and the convergence is actually strong in $W^{1,2}_{loc}$.

From the monotonicity formula for the Ginzburg–Landau energy, $\hat{\mathbf{Q}}_\infty$ is a degree-zero homogeneous harmonic map, hence it is smooth away from the origin by partial regularity theory [34]. Since the latter is constant by Schoen [36], the GL minimizer $\mathbf{Q}_\infty$ is also a constant matrix of norm one from the monotonicity formula for the GL energy. Thus, $|\mathbf{Q}^*_j(0)| \rightarrow |\mathbf{Q}_\infty (0)|=1$ which yields the desired contradiction.

(iv) Let $\delta \in (0,1)$ be fixed. From part (i), (ii) and since ${{\epsilon }}>0$ is fixed and arbitrary, we necessarily have

61 $$\begin{aligned} \beta ^2\left( \mathbf{Q}_j \right) \big |_{\partial B_{{{\epsilon }}}(\mathbf{x}_i)} \le \delta \end{aligned}$$

for *j* sufficiently large, $\mathbf{x}_i \in \Sigma$ (depending only on ${{\epsilon }}$). From (iii) above, we have that $|\mathbf{Q}_j| \rightarrow 1$ uniformly on $\overline{B_{{{\epsilon }}}(\mathbf{x}_i)}$ as $j \rightarrow \infty$.

Thus, if we define the set

62 $$\begin{aligned} \mathcal {N}_\sigma =\left\{ \mathbf{Q}\in S_0 \, \, s.t. \, \, \beta ^2(\mathbf{Q}){\le }\sigma \, \, {\mathrm{and}} \, \, 1-\sigma \le \left| \mathbf{Q}\right| \le 1 \right\} \end{aligned}$$

for each $0\le \sigma <1$ and let $\delta <\sigma$, we have the following:

- The restriction of $\mathbf{Q}_j$ to the boundary, $\mathbf{Q}_j \in C(\partial B_{{{\epsilon }}} (\mathbf{x}_i); \mathcal {N}_\delta )$ for *j* large enough (depending only on ${{\epsilon }}$ [by (61)] and $\mathbf{Q}^0 \in C(\partial B_{{{\epsilon }}} (\mathbf{x}_i); \mathcal {N}_\delta )$ (in view of the inclusion $\mathbf{Q}_{min} =\mathcal {N}_0 \subset \mathcal {N}_\delta$).
- For $\delta<\sigma <1$, the maps $\mathbf{Q}_j$ and $\mathbf{Q}^0$ are homotopic in $C(\partial B_{\epsilon }(\mathbf{x}_i); \mathcal {N}_\sigma )$ (thanks to the uniform convergence; composing pointwise with the affine homotopy in $S_0$ keeps the images inside $\mathcal {N}_\sigma$ for *j* large enough).
- $\mathcal {N}_\sigma \supset \mathcal {N}_0$ retracts homotopically onto $\mathcal {N}_0=\mathbf{Q}_{min}\sim \mathbb {R}P^2$ for every $\sigma <1$, see [8, Lemma 3.10]; see also [10], Corollary 1.2 and Section 5 therein.

Suppose, for a contradiction, that $\max _{\overline{B_{{\epsilon }}(\mathbf{x}_i)}} \beta ^2(\mathbf{Q}_j)<1$ and let $\sigma \in (\max \{\delta , \max _{\overline{B_{{\epsilon }}(\mathbf{x}_i)}} \beta ^2(\mathbf{Q}_j)\} , 1)$. Then the composition of the aforementioned retraction with $\mathbf {Q}_j$ yields a map $\mathbf {Q}_j^* \in C(\overline{B}_{\epsilon }; \mathbf {Q}_{\min })$ whose trace $\mathbf {Q}_j^*|_{\partial B_{\epsilon }}$ is homotopic in $C(\partial B_{\epsilon }; \mathbf {Q}_{\min })$ to $\mathbf {Q}^0|_{\partial B_{\epsilon }}$. By Lemma 3.5 we would conclude that $\deg \mathbf {n}^0|_{\partial B_{\epsilon }}=0$, a contradiction with the fact that ${\text {deg}} ~\mathbf{n}^0|_{\partial B_{\epsilon }}=\pm 1$ near each singular point, for ${\epsilon }$ small enough fixed at the beginning.

Similarly, assume that $\min _{\overline{B_{{{\epsilon }}}(\mathbf{x}_i)}} \beta ^2(\mathbf{Q}_j)>0$ for infinitely many *j* in the sequence. Then $\mathbf{Q}_j(\mathbf{x})$ is purely biaxial for all $\mathbf{x}\in B_{{\epsilon }}(\mathbf{x}_i)$ [recall that there are no isotropic points from part (iii)]. Let $\mathbf {n}_j(\mathbf {x}) \in \mathbb {S}^2$ be the eigenvector corresponding to the maximum eigenvalue (which is uniquely defined up to a sign). Recall that $\mathbf {Q}_j$ is continuous in $\overline{B_{\epsilon }(\mathbf {x}_i)}$, hence $\mathbf {x} \in \Omega \mapsto \mathbf {n}_j \otimes \mathbf {n}_j \in \mathbb {R}P^2$ is continuous (if $\mathbf {x}_k\rightarrow \mathbf {x}$ and $\mathbf {n}_j(\mathbf {x}_k) \overset{k\rightarrow \infty }{\longrightarrow } \mathbf {n}'$ then clearly $\mathbf {n}'$ maximizes $\mathbf {n} \cdot \mathbf {Q}_j(\mathbf {x}) \mathbf {n}$ in $\mathbb {S}^2$ and the maximal eigenvalue is simple). As a consequence, we choose $\mathbf {n}_j$ to be a continuous lifting so that $\mathbf {n}_j \in C(\overline{B_{\epsilon }(\mathbf {x}_i)}; \mathbb {S}^2)$.

Now, $\mathbf {Q}_j$ converges uniformly to $\mathbf {Q}^0$ on $\partial B_{\epsilon }$. Therefore, $|\mathbf {Q}_j| \rightarrow 1$ and $\beta (\mathbf {Q}_j)\rightarrow 0$ uniformly on $\partial B_{\epsilon }$. This implies that $\mathbf {n}_j\otimes \mathbf {n}_j\rightarrow \mathbf {n}^0\otimes \mathbf {n}^0$ uniformly on $\partial B_{\epsilon }$ since

$$\begin{aligned} \left| {\sqrt{\frac{3}{2}}}\left( \mathbf {n}_j\otimes \mathbf {n}_j -\frac{\mathbf {I}}{3}\right) - \frac{\mathbf {Q}_j}{\left| \mathbf {Q}_j\right| }\right| + \left| \frac{\mathbf {Q}_j}{\left| \mathbf {Q}_j\right| } - \mathbf {Q}^0\right| \overset{j\rightarrow \infty }{\longrightarrow } 0. \end{aligned}$$

We conclude that $\mathbf {Q}^0|_{\partial B_{\epsilon }}$ is homotopic to $\sqrt{\frac{3}{2}}(\mathbf {n}_j\otimes \mathbf {n}_j-\frac{\mathbf {I}}{3})$, first in $C(\partial B_{\epsilon }, \mathcal N_\sigma )$ for some small $\sigma$ (composing pointwise with the affine homotopy in $S_0$) and then in $C(\partial B_{\epsilon }, \mathbf {Q}_{\min })$ (composing with the retraction from $\mathcal N_\sigma$ to $\mathbf {Q}_{\min }$). Since $\sqrt{\frac{3}{2}}(\mathbf {n}_j\otimes \mathbf {n}_j-\frac{\mathbf {I}}{3})$ has a continuous extension inside $\overline{B_{\epsilon }}$, we recall Lemma 3.5 and obtain a contradiction with the fact that $\deg \mathbf {n}^0|_{\partial B_{\epsilon }}=\pm 1$ for every ${\epsilon }>0$ small enough.

The uniaxial set has zero Lebesgue-measure, as has already been established in [24, Prop. 14].

(v) For each $\mathbf{x}_i \in \Sigma$ and $\delta \in \left( 0, 1 \right)$ fixed, consider the biaxiality set, $B_{{\epsilon }}(\mathbf{x}_i)\cap B_\delta ^j$, around $\mathbf{x}_i$ and its diameter, $d_j:={{\text {diam}}}\left( B_{{\epsilon }}(\mathbf{x}_i)\cap B_\delta ^j \right)$. We have $d_j=o(1)$ as $j \rightarrow \infty$ from (i) and (ii) above.

We claim that $d_j \sim t_j^{-1/4}$ as $j \rightarrow \infty$, which follows by blowing up $\mathbf{Q}_j$, at scale $d_j$, and excluding remaining decay rates. Firstly, let $\mathbf {p}_j, \mathbf {q}_j \in B_{{\epsilon }}(\mathbf{x}_i)\cap B_\delta ^j$ such that $d_j=|\mathbf {p}_j-\mathbf {q}_j|$ and let $\hat{\mathbf{x}}_j:=(\mathbf {p}_j+\mathbf {q}_j)/2$. Clearly $\left( B_{{\epsilon }}(\mathbf{x}_i)\cap B_\delta ^j \right) \subseteq B(\hat{\mathbf{x}}_j, \frac{3d_j}{2})$ and $\hat{\mathbf{x}}_j \rightarrow \mathbf{x}_i$ as $j \rightarrow \infty$. Then by defining $B^j:=B^j(\hat{\mathbf{x}}_j,d_j/2)$ and by (ii), we immediately have $\beta ^2\left( \mathbf{Q}_j \right) \le \delta$ on $\partial B(\hat{\mathbf{x}}_j, \frac{3d_j}{2})$, $\beta ^2\left( \mathbf{Q}_j \right) = \delta$ at two antipodal points on $\partial B^j$, and $\displaystyle \max _{\overline{B}(\hat{\mathbf{x}}_j, \frac{3d_j}{2})}\beta ^2\left( \mathbf{Q}_j \right) =1$, for *j* large enough.

Define $\hat{\mathbf{Q}}_j(\mathbf{x}) = \mathbf{Q}_j\left( \hat{\mathbf{x}}_j + d_j \mathbf{x}/2 \right)$ and we get, up to a sequence of rotations which we do not specify explicitly,

63 $$\begin{aligned} \beta ^2\left( \hat{\mathbf{Q}}_j \right) |_{\partial \left( \frac{3}{2}B\right) } \le \delta \, , \quad \beta ^2\left( \hat{\mathbf{Q}}_j (0,0,\pm 1) \right) = \delta \, , \quad \max _{\overline{\left( \frac{3}{2}B\right) }}\beta ^2\left( \hat{\mathbf{Q}}_j \right) = 1 \end{aligned}$$

on the unit ball $B = B\left( 0,1 \right)$. The the rescaled maps $\hat{\mathbf{Q}}_j$ are defined on the family of expanding domains, $2(\Omega - \hat{\mathbf{x}}_j )/ d_j \rightarrow {\mathbb {R}}^3$ and are local minimizers on compact subdomains of the functionals

64 $$\begin{aligned} I_j\left[ \hat{\mathbf{Q}}_j \right] : = \int \frac{\bar{L}}{2}\left| {{\nabla }}\hat{\mathbf{Q}}_j\right| ^2 + \frac{d^2_j}{4} \left[ \frac{t_j}{8}\left( 1 - \left| \hat{\mathbf{Q}}_j\right| ^2 \right) ^2 + \frac{h_+}{8}\left( 1 + 3\left| \hat{\mathbf{Q}}_j\right| ^2 - 4\sqrt{6}{\text {tr}}\hat{\mathbf{Q}}_j^3 \right) \right] ~dV \end{aligned}$$

with $h_+ \sim \sqrt{t_j}$ as $j \rightarrow \infty$. Taking into account the Euler–Lagrange equations [corresponding to (64)], we can exclude the following regimes:

(a) $d_j<< t_j^{-1/2}$ since we easily deduce that (up to subsequences) $\hat{\mathbf{Q}}_j \rightarrow \mathbf{Q}_*$ in $C^k_{loc}\left( {\mathbb {R}}^3 \right)$ for $k \in \mathbb {N}$ by the uniform $L^\infty$-bound and elliptic regularity. Indeed, for $d_j<< t_j^{-1/2}$, the nonlinear terms in the Euler–Lagrange equations vanish as $j \rightarrow \infty$. Thus $\mathbf{Q}_* \in C^2\left( {\mathbb {R}}^3 \right)$ is bounded and harmonic, hence constant (of norm one from (iii) above) by Liouville’s Theorem and this fact contradicts (63) which holds for the limiting map $\mathbf{Q}_*$ by uniform convergence.

(b) $$d_j \sim t_j^{-1/2}$$ ; this regime has already been discussed in item (iii) above (when proving norm convergence generalizing results in [10]) and hence, up to a subsequence, $$\hat{\mathbf{Q}}_j \rightarrow \mathbf{Q}_{**}$$ in $$C^k_{loc}\left( {\mathbb {R}}^3 \right) $$ for $$k \in \mathbb {N}$$. Here $$\mathbf{Q}_{**}$$ is a bounded Ginzburg–Landau local minimizer on the whole of $$\mathbb {R}^3$$ such that $$\int _{B_R} \frac{1}{2}|{{\nabla }}\mathbf{Q}_{**}|^2 + \left( 1 - |\mathbf{Q}_{**}|^2 \right) ^2 =\mathcal {O} (R)$$ as $$R \rightarrow \infty $$. Arguing as in Lemma 3.6 and item (iii) above, we infer that $$\mathbf{Q}_{**}$$ is a constant matrix of norm one, contradicting (63) which still passes to the limit under smooth convergence and clearly cannot hold for constant maps.

(c) $t_j^{-1/2}<< d_j<< t_j^{-1/4}$. Here (up to a subsequence), the limiting map is a local minimizer of $\int |{{\nabla }}\mathbf{Q}|^2$ among $\mathbb {S}^4$-valued maps. Indeed the sequence is locally bounded in $H^1_{\mathrm{loc}}(\mathbb {R}^3)$ by the monotonicity formula and hence converges weakly in $H^1_{\mathrm{loc}}$ (up to a subsequence). The limiting map is clearly $\mathbb {S}^4$-valued, as can be seen by applying Fatou’s Lemma to (64). Additionally, we can prove strong convergence to the limiting map and the minimality of the limiting map, arguing as in item (iii) above, exactly as in Lemma 4.1 of [10]. We omit the details for brevity.

Therefore, $\hat{\mathbf{Q}}_j \rightarrow \mathbf{Q}_h$ in $H^1_{loc}\left( {\mathbb {R}}^3 \right)$ and $\mathbf{Q}_h \in W^{1,2}_{loc}\left( {\mathbb {R}}^3, \mathbb {S}^4 \right)$ is a locally minimizing harmonic map. At this point, we appeal to two powerful and celebrated results in [36] (also see [34]) (i) If $n \le d(k)$, then every minimizing map from a manifold *M* of dimension *n* into $S^k$ is smooth in the interior of *M* and in our case $n=3, k=4$ and $d(4) = 3$ and (ii) for $k > 3$ and $n \le d(k)$, there is no non-constant minimizing map *u* from ${\mathbb {R}}^n$ into $S^k$. This shows that $\mathbf{Q}_h \in C^\infty (\mathbb {R}^3; \mathbb {S}^4)$ by the constancy of stable tangent maps into spheres proven in [36]. We use the constancy of stable tangent maps into spheres from [36] and the monotonicity formula, arguing by analogy with case (b), to infer that $\mathbf{Q}_h$ is a constant matrix of norm one. In view of this constancy property, we can improve the convergence $\hat{\mathbf{Q}}_j \rightarrow \mathbf{Q}_h$ in $H^1_{loc}\left( {\mathbb {R}}^3 \right)$ to a smooth convergence [we just need to use the argument based on the Bochner inequality from (i) above]. Since biaxiality is constant for constant maps, we contradict (63).

(d) Finally, we consider the regime $t_j^{-1/4}<< d_j<< 1$. Here, the limiting energy is again the Dirichlet energy, $\int |{{\nabla }}\mathbf{Q}|^2~dV$, for $\mathbf{Q}_{min}$-valued maps in $H^1_{\mathrm{loc}}(\mathbb {R}^3)$, as can be seen by applying Fatou’s Lemma to (64). We again have $\hat{\mathbf{Q}}_j \rightarrow \mathbf{Q}_h$ in $H^1_{loc}\left( {\mathbb {R}}^3 \right)$, arguing similarly to part (c) above. However, from the uniaxiality of the limiting tensor and the lifting results in [3], we lift $\mathbf{Q}_h$ to an $\mathbb {S}^2$-valued minimizing harmonic map $\bar{\mathbf {n}} \in H^1_{loc}(\mathbb {R}^3;\mathbb {S}^2)$. From the classification result for harmonic unit-vector fields, such as $\bar{\mathbf {n}}$, in [1, Thm. 2.2], we either have $\mathbf{Q}_h = constant$ or $\mathbf{Q}_h = \sqrt{\frac{3}{2}}\left( \frac{\mathbf{x}\otimes \mathbf{x}}{|\mathbf{x}|^2} - \frac{\mathbf {I}}{3} \right)$. This is in contrast to case (c) above where all minimizing maps from ${\mathbb {R}}^3$ to $S^4$ are constant. Again as in step (c), we can improve the convergence $\hat{\mathbf{Q}}_j \rightarrow \mathbf{Q}_h$ in $H^1_{loc}\left( {\mathbb {R}}^3 \right)$ to locally smooth convergence except at most at one point (combining the smoothness of the limiting map with small energy regularity to infer smooth convergence). This contradicts (63) since $\beta ^2\left( \mathbf{Q}_h \right) = 0$ everywhere except possibly for the origin, since $\mathbf{Q}_h$ is uniaxial for $\mathbf{x}\ne \mathbf {0}$. $\square$

### Proof of Theorem 2

We can prove the existence of a global LdG minimizer $\mathbf{Q}_j$, of the re-scaled energy (17), in the restricted class of uniaxial $\mathbf{Q}$-tensors, for each $t_j$, from the direct methods in the calculus of variations. It suffices to note that the uniaxiality constraint, $6\left( {\text {tr}} \mathbf{Q}^3 \right) ^2 = |\mathbf{Q}|^6$ is weakly closed and the existence result follows immediately.

The limiting harmonic map $\mathbf{Q}^0$ is uniaxial and hence, the energy bound (22) follows immediately since the upper bound is simply the re-scaled LdG energy of $\mathbf{Q}^0$. The uniaxial map, $\mathbf{Q}_j = s_j\left( \mathbf{n}_j \otimes \mathbf{n}_j - \frac{\mathbf {I}}{3} \right)$, necessarily has non-negative scalar order parameter, $s_j$. Indeed, note that by uniaxiality, $\det \, \mathbf{Q}(x) >0$ (resp. $\det \, \mathbf{Q}(x)<0$) iff $\mathbf{Q}(x)$ has positive (resp. negative) scalar order parameter and also that $\det \, \mathbf{Q}(x)=0$ iff $\mathbf{Q}(x) =0$ at any $x\in \Omega$. We set $\Omega _j:= \{ \det \, \mathbf{Q}_j(x)<0 \} \subset \Omega$, which is an open subset (possibly empty), since $\mathbf{Q}_j$ is globally Lipschitz in $\Omega$. If $\Omega _j \ne \emptyset$, then we define the uniaxial admissible perturbation

65 $$\begin{aligned} \mathbf{Q}_j^* (\mathbf{r}) = {\left\{ \begin{array}{ll} \mathbf{Q}_j \qquad \mathbf{r}\in \Omega \setminus \Omega _j \\ - \mathbf{Q}_j \quad \mathbf{r}\in \Omega _j \end{array}\right. } \end{aligned}$$

and one can easily check that $\mathbf{Q}^*_j$ is globally Lipschitz in $\Omega$ and $\frac{3\bar{L}}{2Ls_+^2 D} \mathbf {I}^j_{LG}[\mathbf{Q}_j^*] < \frac{3\bar{L}}{2Ls_+^2 D} \mathbf {I}^j_{LG}[\mathbf{Q}_j]$, contradicting the assumed global minimality of $\mathbf{Q}_j$ in the restricted class of uniaxial $\mathbf{Q}$-tensors. We can then appeal to Proposition 2.1 and proceed by contradiction. We assume that the global LdG-minimizers, $\mathbf{Q}_j$, in the restricted class of uniaxial $\mathbf{Q}$-tensors, are stable critical points of the LdG energy, for *j* large enough. The sequence, $\left\{ \mathbf{Q}_j \right\}$, then satisfies the hypothesis of Proposition 2.1, for large *j*. We thus, conclude that each $\mathbf{Q}_j$, has a set of isotropic points $\mathbf{x}_i^{(j)}$ (at least one near each singular point $\mathbf{x}_i$ of $\mathbf{Q}^0$) and $\mathbf{Q}_j$ is asymptotically described by the RH-profile near each isotropic point $\mathbf{x}_i^{(j)}$ as $j \rightarrow \infty$ in the sense of Proposition 2.1. Recall that the RH-solution, (23) is known to be unstable with respect to biaxial perturbations localized around the origin [22, 26], for large *t*. This suffices to prove that global minimizers in the restricted class of uniaxial $\mathbf{Q}$-tensors cannot be stable critical points of the LdG energy in this limit, since stability of $\mathbf{Q}_j$ would pass to the limit under smooth convergence. $\square$

### Proof of Proposition 2.1

*Proof of (i):* By Propositions 3.1 and 3.2, after extracting a subsequence, we have that $\left\{ \mathbf{Q}_j\right\}$ converges strongly in $W^{1,2}$ and uniformly away from the singular set $\Sigma =\left\{ \mathbf{x}_1 \ldots \mathbf{x}_N \right\}$, to a (minimizing) limiting harmonic map, $\mathbf{Q}^0$. We prove that for each $i=1,\ldots , N$ and every fixed $r_0>0$ sufficiently small, there exists $j_0\in \mathbb {N}$ such that for every $j\ge j_0$, the map $\mathbf {Q}_j$ has an isotropic point, $\mathbf {x}_i^{(j)}$, in $\overline{B}(\mathbf {x}_i, r_0)$. The stated conclusion then follows by a diagonal argument on $r_0$. Suppose, for a contradiction, that we can find a subsequence, $\{j_k\}_{k\in \mathbb {N}}$, such that $\min _{B(\mathbf {x}_i, r_0)} |\mathbf {Q}_{j_k}|>0$ for all $k\in \mathbb {N}$. Since $\mathbf {Q}_j$ is purely uniaxial for all *j* by assumption, we have that $\frac{\mathbf {Q}_{j_k}}{|\mathbf {Q}_{j_k}|}$ is continuous on $\overline{B(\mathbf {x}_i, r_0)}$ and the uniform convergence to $\mathbf {Q}^0$ implies that $\frac{\mathbf {Q}_{j_k}}{|\mathbf {Q}_{j_k}|}$ converges uniformly to $\mathbf {Q}^0$ on $\partial B_{\epsilon }$. Arguing as in the proof of Theorem 1 (iv) we obtain a contradiction.

*Proof of (ii):* The aim is to prove that $\mathbf {Q}_j$ has a RH type of profile, (23), near each singular point in $\Sigma$, for *j* sufficiently large. The proof follows from Lemma 3.6 and Propositions 4 and 8 in [15]. We begin by noting that for each $i=1\ldots N$ in $\{\mathbf {x}_1, \ldots , \mathbf {x}_N\}$, we can extract a sequence, $\left\{ \mathbf {x}_j^*\right\}$, such that $\mathbf {Q}_j\left( \mathbf {x}_j^* \right) = 0$ and $\mathbf {x}_j^* \rightarrow \mathbf {x}_i$ as $j\rightarrow \infty$. By Lemma 3.6, the rescaled maps (51) converges in $\bigcap _{k\in \mathbb {N}} C^{k}_{{{\mathrm{loc}}}}$ to a classical solution ${\tilde{\mathbf {Q}}}^\infty$ of the Ginzburg–Landau equations satisfying the energy growth (52). Moreover, it can be seen that ${\tilde{\mathbf {Q}}}^\infty$ is uniaxial and has a non-negative scalar order parameter. Finally ${\tilde{\mathbf {Q}}}^\infty (\mathbf {0})=\mathbf {0}$ because ${\tilde{\mathbf {Q}}}_{j_k}(0)=\mathbf {0}$ for each *k*, by assumption. We conclude that the hypotheses of [15, Prop. 8] are satisfied. We reproduce the statement of [15, Prop. 8] below, for completeness. $\square$

### Proposition 3.7

(Proposition 8, [15]) Let $\mathbf {Q}\in C^2(\mathbb {R}^3; S_0)$ be a uniaxial solution of $\Delta \mathbf{Q}= (|\mathbf{Q}|^2-1)\mathbf{Q}$ with $\mathbf {Q}(0)=0$ and non-negative scalar order parameter, satisfying the energy bound (52). Let *h* denote the unique solution for the boundary-value problem (24). Then there exists an orthogonal matrix $\mathbf {T} \in \mathcal O(3)$ such that

66 $$\begin{aligned} \mathbf{Q}(\mathbf {x}) = \sqrt{\frac{3}{2}} h( \left| \mathbf{x}\right| ) \left( \frac{\mathbf {T} \mathbf{x}\otimes \mathbf {T}\mathbf{x}}{\left| \mathbf{x}\right| ^2} - \frac{\mathbf {I}}{3} \right) , \quad \mathbf{x}\in \mathbb {R}^3. \end{aligned}$$

This yields the conclusion of Proposition 2.1. $\square$

## Conclusions

Theorem 1 focuses on global LdG minimizers on arbitrary 3D domains, with arbitrary topologically non-trivial Dirichlet conditions, subject to fixed $\bar{L}$ in (17), in the $t\rightarrow \infty$ limit. We prove that global minimizers are well described by a (minimizing) limiting harmonic map, $\mathbf{Q}^0$, away from the singular set of $\mathbf{Q}^0$. Further, we prove that the norm of a global minimizer converges uniformly to a constant everywhere and there is at least a point of maximal biaxiality (with $\beta ^2=1$) and a point of uniaxiality (with $\beta ^2=0$) near each singular point of $\mathbf{Q}^0$, in this asymptotic limit. As explained in the introduction, these results provide a rigorous justification for the widely used Lyuksyutov constraint for low temperatures [17, 18]. Further, they give us quantitative information about the expected number and location of points (or regions) of maximal biaxiality, which was previously not proven in the literature. In other words, a precise knowledge of the limiting map $\mathbf{Q}^0$ yields accurate information about both the far-field behaviour, the location of the defect cores and some partial information about the structure of the defect cores, which appears to be independent of the geometry. Numerical results in [17, 18] support our analysis that defect structures are independent of the geometry for “large” domains.

We briefly explain how our results are related to the celebrated biaxial torus structure of nematic defect cores reported in [14, 17, 32, 37]. The biaxial torus has been largely reported in 3D droplets although one could conjecture that it is a generic defect structure independent of geometry. The biaxial torus usually describes a uniaxial ring of negative scalar order parameter enclosed by a torus of maximal biaxiality, as has been suggested by the exhaustive work in [14, 17, 32, 37]. There is no rigorous proof of the existence of such a biaxial torus and our results are only a first step in that direction. In Theorem 1, we prove the co-existence of maximal biaxiality and uniaxiality near each singular point of $\mathbf{Q}^0$, which is at least consistent with the biaxial torus picture. Indeed, rigorous analytical results such as ours could motivate experimentalists to probe into defect cores, in quest of the biaxial torus or indeed other defect structures, which may have the qualitative properties proven in Theorem 1.

Theorem 2 is a statement on global LdG minimizers of (17) within the restricted class of uniaxial $\mathbf{Q}$-tensors in the $t \rightarrow \infty$ limit. These constrained uniaxial minimizers exist although they need not be critical points of the LdG energy. Indeed, uniaxial solutions of (15) are, in general, difficult to find but the RH solution is a 3D uniaxial critical point of the LdG energy i.e. is a solution of the system (15) of the form (7) with $s>0$ for $r>0$ on a 3D droplet [14, 15, 20]. Indeed, one could imagine a continuous uniaxial perturbation of the RH solution that remains a solution of the system (15), at least in an approximate sense which needs to be carefully defined. In the absence of a rigorous exclusion of generic uniaxial critical points in 3D, Theorem 2 and Proposition 2.1 have a twofold purpose: (i) firstly, they rule out the stability of such uniaxial critical points, if they can be constructed and (ii) secondly, they establish the universal RH-type defect profiles for such uniaxial critical points with specific properties as in Theorem 2 (if they exist). We hope to make rigorous studies of uniaxial and biaxial defect profiles, in differents asymptotic limits, in future work.
